# Biological function analysis of ARHGAP39 as an independent prognostic biomarker in hepatocellular carcinoma

**DOI:** 10.18632/aging.204635

**Published:** 2023-04-05

**Authors:** Yongqi Ding, Yiyang Gong, Hong Zeng, Xuanrui Zhou, Zichuan Yu, Jingying Pan, Minqin Zhou, Shiwen Liu, Wei Lai

**Affiliations:** 1 Department of Health Management Medical, The Second Affiliated Hospital of Nanchang University, Nanchang, China; 2 Second College of Clinical Medicine, Nanchang University, Nanchang, China; 3 Emergency Intensive Care Unit, The First Affiliated Hospital of Nanchang University, Nanchang, China

**Keywords:** ARHGAP39, biomarker, hepatocellular carcinoma, prognosis, immune infiltration

## Abstract

Hepatocellular carcinoma (HCC) is the most common subtype of liver cancer, with a high morbidity and low survival rate. Rho GTPase activating protein 39 (ARHGAP39) is a crucial activating protein of Rho GTPases, a novel target in cancer therapy, and it was identified as a hub gene for gastric cancer. However, the expression and role of ARHGAP39 in hepatocellular carcinoma remain unclear. Accordingly, the cancer genome atlas (TCGA) data were used to analyze the expression and clinical value of ARHGAP39 in hepatocellular carcinoma. Further, the LinkedOmics tool suggested functional enrichment pathways for ARHGAP39. To investigate in depth the possible role of ARHGAP39 on immune infiltration, we analyzed the relationship between ARHGAP39 and chemokines in HCCLM3 cells. Finally, the GSCA website was used to explore drug resistance in patients with high ARHGAP39 expression. Studies have shown that ARHGAP39 is highly expressed in hepatocellular carcinoma and relevant to clinicopathological features. In addition, the overexpression of ARHGAP39 leads to a poor prognosis. Besides, co-expressed genes and enrichment analysis showed a correlation with the cell cycle. Notably, ARHGAP39 may worsen the survival of hepatocellular carcinoma patients by increasing the level of immune infiltration through chemokines. Moreover, N6-methyladenosine (m^6^A) modification-related factors and drug sensitivity were also found to be associated with ARHGAP39. In brief, ARHGAP39 is a promising prognostic factor for hepatocellular carcinoma patients that is closely related to cell cycle, immune infiltration, m6A modification, and drug resistance.

## INTRODUCTION

Evidence disclosed that liver cancer ranks as the 5th most common cancer throughout the world, and occupies the second spot in the cancer mortality rates [1]. Hepatocellular carcinoma (HCC) is a primary liver tumor that accounts for more than 90% of all types of primary liver tumor, with high rates of metastasis, recurrence, and mortality [2], as well as poor prognosis [3]. Viral hepatitis, including hepatitis B and C, alcoholic liver disease, and nonalcoholic fatty liver disease, is a risk factor for liver cancer, and up to 85% of hepatocellular carcinomas occur in patients with cirrhosis [4]. At present, HCC can be diagnosed through laboratory testing of serum biomarkers (alpha-fetoprotein, AFP) and imageological examination (including ultrasound, CT/MRI imaging, and biopsy), but most HCC patients are diagnosed late and miss the best treatment time [5]. Although, for the past few years, some progress has been made in the treatment of HCC, the survival and recovery rate of patients are still not optimistic, with only an approximately 18% five-year survival rate [6–8]. As biochemical indicators reflecting changes in the structure or function of human organs, biomarkers are often used in the diagnosis and staging of diseases or to evaluate the effectiveness of drugs and treatments. Clinical biomarkers for HCC are mainly serum biomarkers, like alpha-fetoprotein (AFP). Other biomarkers include DES - γ -carboxyprothrombin (DCP) and lectin binding α -fetoprotein, which may be elevated in HCC [9]. However, these biomarkers are still not perfect for further effective diagnosis and treatment. Therefore, to increase the diagnostic efficiency of HCC and optimize the therapeutic effect, it has become an urgent task to explore effective biomarkers for HCC.

HCC is a complex ecosystem containing different kinds of immune-related cells. The successful use of immune checkpoint inhibition in tumors has confirmed the critical role of the tumor microenvironment in tumor development [10]. Approximately 30% of early HCC have genomic evidence of immune activation, while 25% have no immune infiltration [11]. Studying the tumor microenvironment is crucial for developing new therapies and identifying biomarkers [12].

ARHGAP39, also known as preoptic regulatory factor-2 (Porf-2) or Vilse, is a member of the Rho GAP group and a Rho GTP-activating protein that plays an important role in neural development [13]. It’s known to modulate apoptosis, cell migration, neurogenesis, and the morphology of dendritic spines in the brain and hippocampus. What has also been reported is that ARHGAP39 is able to inhibit the proliferation of neural stem cells (NSC) via enhancing the level of P21 protein or play a pro-apoptotic role in drug therapy through p53 transcription-dependent and independent pathways [14]. ARHGAP39 is widely distributed in various parts of the body and has a potential role in tumorigenesis [15]. Mutations, copy number variants (CNVS), or expression levels of ARHGAP39 have been observed in several kinds of phymatoid tissues in the central nervous system, skin, prostate, and gastrointestinal tract [16]. It has also been established that ARHGAP39 interacts with p53 and BAX, and that when ARHGAP39 is down-regulated, cell proliferation can be promoted, potentially leading to tumorigenesis [14]. Meanwhile, ARHGAP39 has the function of activating Rho GTPase, while Rho GTPase is already known to participate in cytodynamics, cell growth, cell intimal transport, as well as apoptosis [17], which is identified as a new target in oncotherapy [18]. As a result, studying the expression and related mechanisms of ARHGAP39 in cancers is likely to be extremely beneficial to cancer treatment. We find that ARHGAP39 has not been reported in HCC, and its relationship with prognosis remains unclear.

Among our research, our group studied the function of ARHGAP39 in HCC from the expression level of ARHGAP39, survival analysis, the relationship between ARHGAP39 and cell cycle, tumor-infiltrating immune cells, m^6^A modification, drug sensitivity, and so on. It also provides a potential link between ARHGAP39 and the cell cycle, m^6^A modification, drug sensitivity, HCC immune invasion, and its underlying mechanisms.

## MATERIALS AND METHODS

### Data collection and processing

ARHGAP39 expression data were derived from the liver hepatocellular carcinoma (LIHC) dataset in the TCGA database (https://portal.gdc.cancer.gov), which is made up of 374 LIHC samples and 50 normal tissues (Workflow Type: HTSeq-FPKM), and the Liver Cancer - NCC, and JP datasets in the ICGC database (https://dcc.icgc.org), which are composed of 202 normal samples and 243 tumor samples. LIHC clinical information was derived from the TCGA database, which contained 377 samples.

### Cell culture

Human HCC cell line LM3 was transfected in 37°C DMEM (HyClone, Germany) and 10% fetal bovine serum (Gibco, USA). HCCLM3 was obtained from the Cell Bank of Type Culture Collection of the Chinese Academy of Sciences and the Shanghai Institute of Cell Biology in China.

### TIMER database analysis

TIMER (https://cistrome.shinyapps.io/timer/) [19], a website designed to explore the invasion of immune cells in tumor tissue, was applied to check into the association between ARHGAP39 expression and the infiltration level of various immune cells in HCC, especially T cells. This research chose the “Diff Exp module” for exploring the ARHGAP39 expression in certain tumors, and the “Gene module” for analyzing the connection between ARHGAP39 expression and the infiltration level of immune cells in specific cancers. In addition, with the help of the “Correlation module”, considering the Spearman’s rho value (*p* value < 0.05) and predicted statistical implications, the relationship between the expression level of ARHGAP39 and that of the immune cell markers in HCC was studied. Furthermore, we dug into the relationship between ARHGAP39 and immune checkpoint genes at the level of expression through the “Correlation module”.

### HCCDB database

HCCDB (http://lifeome.net/database/hccdb) [20] designed for exploring HCC, covers 15 public HCC gene expression datasets from 3917 samples. What’s more, the expression pattern of each gene can be studied based on the data from TCGA and Genotype-Tissue Expression (GTEx). Simultaneously, HCCDB provides links to third-party databases and shows the results graphically. Through the website, survival analysis was performed and co-expression networks of HCC tissues were constructed. *p* value < 0.05 was considered meaningful.

### UALCAN database analysis

UALCAN (http://ualcan.path.uab.edu/index.html) [21] is a bioinformatics tool that includes gene expression and clinic pathology data from the TCGA database. In our research, we utilized UALCAN to figure out the relationships between ARHGAP39 expression and clinical parameters. Furthermore, we explored the survival of different cohorts stratified by gender, weight, and grade. We further studied the level of ARHGAP39 promoter methylation.

### Kaplan-Meier plotter database analysis

Kaplan-Meier plotter (https://kmplot.com/analysis/) [22] is a full-scale website that was employed to evaluate the prognostic value of genes in all carcinomas. In the research, four survival outcomes of ARHGAP39 were downloaded, including overall survival (OS), progression-free survival (PFS), recurrence-free survival (RFS), and disease-specific survival (DSS). Simultaneously, the expression, OS, and RFS of ten potential hub genes were downloaded. Subsequently, we downloaded several cytokines for survival in HCC. Ultimately, the association between the HCC patients’ survival and various immune cells was explored. The HR with a 95% CI was marked.

### MEXPRESS

MEXPRESS (https://mexpress.be) [23, 24] is an accessible website visualizing DNA methylation levels. We input “ARHGAP39” to investigate the DNA methylation levels of ARHGAP39 in HCC.

### SMART

Shiny methylation analysis resource tool (http://www.bioinfo-zs.com/smartapp/) [25] is a convenient resource that thoroughly deals with the DNA methylation data derived from TCGA. We attempted to find the position distribution on the chromosomes of several CpG sites by inputting “ARHGAP39”.

### LinkedOmics

LinkedOmics (http://www.linkedomics.org/login.php) [26], an online analysis site, integrates global proteomics data grounded on TCGA tumor samples, is usually used to analyze multidimensional data within and across 32 kinds of cancer. Using the “LinkFinder module”, the co-expressed genes linked to ARHGAP39 in the TCGA-LIHC database were visualized with volcano plots and heat maps. In addition, in the “LinkInterpreter module”, we made Gene Ontology (GO) and Kyoto Encyclopedia of Genes and Genomes (KEGG) analyses to seek functional enrichment of ARHGAP39-correlated genes. What’s more, we sought pathways with important biological roles in cancer occurrence and progression via the “LinkInterpreter module” in “GSEA” mode. Pathways with *p* value < 0.05 as the standard.

### PPI network construction

STRING (https://string-db.org/) [27] is designed for exploring associations between all known and predicted proteins, including physical interactions and functional associations. We built a Protein-Protein Interaction Network (PPI) with the top 500 genes most closely related to ARHGAP39, chosen from volcano plots. Using it, we studied the connection among these genes. The parameter of medium confidence was set at 0.4. The top 500 genes were evaluated by Cytoscape 3.9.0 with the MCC method. And the selection criteria are as follows: Max depth = 100, node score cutoff = 0.2, K-core = 2.

### GEPIA analysis

GEPIA (http://gepia.cancer-pku.cn/detail.php) [28], an integrated online resource based upon the TCGA database, was utilized to assess ARHGAP39 expression in “Expression DIY” module and analyze the associations between ARHGAP39 and immune checkpoints in the “correlation analysis” module, namely CD274, CTLA4, CCR8, HAVCR2, TGFB1, and STAT5B, respectively. What’s more, the survival of m^6^A-related genes was investigated. The spearman correlation coefficient was employed to assess their relationships in HCC.

### TISIDB analysis

The TISIDB database (http://cis.hku.hk/TISIDB/index.php) [29] is an interactive website collecting massive tumor data from the TCGA database. The “Chemokine” module was used to explore correlations between ARHGAP39 and cytokines. Especially, the scatter diagrams, representing the correlation between CCL20 and CXCL1 and ARHGAP39 expression, were investigated. Meanwhile, differential expression of ARHGAP39 in various immune subtypes of cells in HCC was also found.

### GeneMANIA analysis

GeneMANIA (http://www.genemania.org) [30] is a one-of-a-kind online resource for gene function and lists analyses. We used it to draw an interactive functional network for ARHGAP39. In the network, we used lines of various thicknesses and colors to show the functional relationship and correlation strength between the two connected ends.

### Protein structure and docking analysis

cBioPortal (http://cbioportal.org) [31] was employed to analyze the secondary structures of ARHGAP39, SLIT2, and ROBO1 with the sample (study ID, LIHC-TCGA-Firehouse Legacy). We penetrating SLIT2 and ROBO1 advanced structures from the PDB database (https://www.rcsb.org/) (PDB ID: 2WFH and 5O5I) [32]. Besides, the SWISS-MODEL Database (https://swissmodel.expasy.org/) predicted the advanced structure of ARHGAP39 (SWISS-MODEL ID: Q9C0H5) [33]. Ultimately, the interaction docking patterns between ARHGAP39 and SLIT2 and ROBO1 were predicted by the HDOCK server (http://hdock.phys.hust.edu.cn/) and visualized utilizing PyMOL software [34].

### Cancer pathway activity and drug sensitivity

GSCALite (http://bioinfo.life.hust.edu.cn/web/GSCALite/) [35] integrates large amounts of multiomics and drug data to assess a series of genes in cancer. Using the TCGA LIHC dataset, it was used to analyze drug sensitivity and cancer pathway associated with ARHGAP39 expression.

### CTD analysis

CTD (http://ctdbase.org/) [36] is a reformatory database, providing toxicological information for chemicals, genes, phenotypes, and diseases. We used it to gather information about chemicals and drugs that may work to regulate ARHGAP39 expression. And with these results, we constructed the ARHGAP39–drug interaction network.

### Quantitative RT-PCR analysis

The standard Trizol-based protocol (Invitrogen, USA) was used to extract total mRNA, and the PrimeScript RT Reagent Kit (Invitrogen, USA) performed a reverse transcription reaction. Then qPCR was conducted by SYBR Premix Ex Taq (TaKaRa, China). Finally, semi-quantitative analysis was performed. This technique was adopted in our study to examine the relative mRNA expression of CCL20 and CXCL1.

### Statistical analysis

R 4.1.2 software was used for statistical analyses. The discrepancy in ARHGAP39 expression between HCC samples and normal samples was reflected by adopting “limma” and “bee swarm” packets of “R” and rank sum test. Logistic regression was employed to assess the association between ARHGAP39 and clinicopathological characteristics. The Kaplan-Meier curve revealed the prognosis distribution among patients with different expressions. Univariate and Multivariate Cox regression analysis identified factors connected with prognosis (*p* < 0.05) (the Cox model uses the “survival” and “survminer” packages of “R”). The ROC curve drawn by “survival ROC” was applied to analyze the predictive capacity of ARHGAP39 expression levels for 1-, 3-, and 5-year survival. Heat maps and scatter plots showing the relationship between ARHGAP39 and m^6^A-related genes and a Venn diagram were made with “ggplot2”.

### Data availability statement

Publicly available datasets were analyzed in this study. The data are accessible in TCGA and ICGC databases. Further inquiries can be directed to the corresponding author.

## RESULTS

### ARHGAP39 is over-expressed in HCC

**Figure 1:**
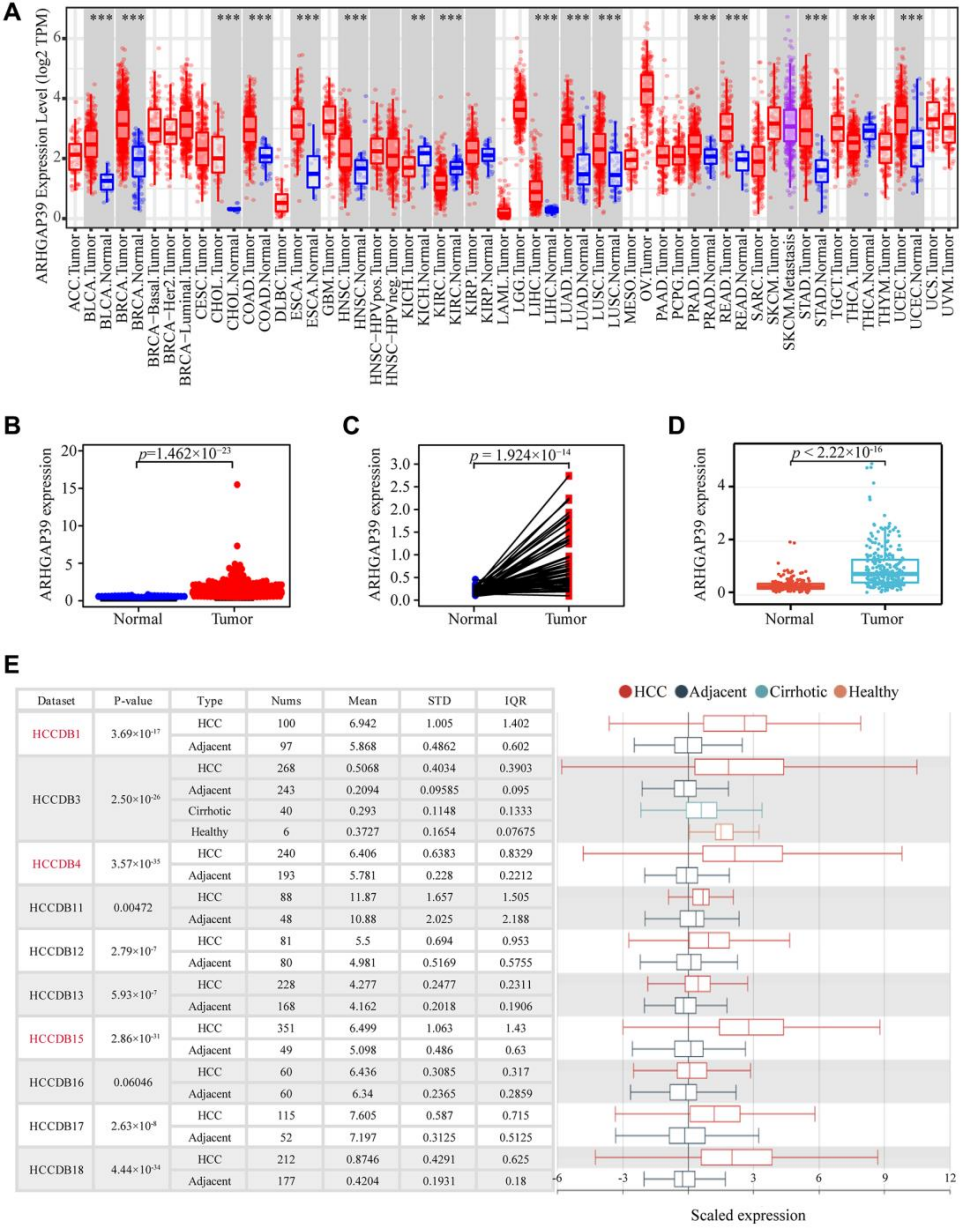
**The expression of ARHGAP39 in HCC and other cancers.** (**A**) Level of ARHGAP39 expression in a variety of cancer tissues (^***^p < 0.001, ^**^p < 0.01, ^*^p < 0.05). (**B**) ARHGAP39 mRNA levels in tumor and normal tissues based on the TCGA database (*p* = 1.462e−23). (**C**) Paired differential expression map of ARHGAP39 between HCC tissues and normal tissues based on the TCGA database (*p* = 1.924e−14). (**D**) The mRNA expression level of ARHGAP39 in tumor and normal tissues in the ICGC (*p* < 2.22e−16). (**E**) HCCDB analysis of aberrant expression of ARHGAP39 in HCC patients.

To elucidate the association between ARHGAP39 expression and HCC, we explored the mRNA levels of ARHGAP39 in the TCGA and TIMER databases. The high expression of ARHGAP39 mRNA was detected in 13 types of cancer, including LIHC (Figure 1A). Further, scatter and paired diagrams revealed that the tumor expression level was significantly higher than para-carcinoma tissues in LIHC (Figure 1B, 1C). Meanwhile, the mRNA level of ARHGAP39 in ICGC databases also showed the same result (Figure 1D). Additionally, another online website, HCCDB, was also utilized to investigate that. We could observe that nine data sets were significant, verifying previous results (Figure 1E). To sum up, these results suggested that ARHGAP39 was overexpressed in HCC.

### Connection between ARHGAP39 expression and clinical characteristics features of HCC patients

We measured the levels of ARHGAP39 expression in different cohorts based on age, gender, tumor grade and stage, and T and N classification to validate the link between ARHGAP39 and multiple clinic pathological features. The results indicated that ARHGAP39 expression was related to age, histologic grade, stage, and T classification (Supplementary Figure 1A–1F, *p* < 0.05**)**, while there was no significant link with gender and N classification. Furthermore, logistic regression analysis revealed that ARHGAP39 expression was associated with pathological grade (grade III vs. I, *p* = 0.041), stage (stage II vs. I, *p* = 0.001), and T classification (T2 vs. T1, *p <* 0.001) (Supplementary Table 1). Further, we also checked through the UALCAN website (Supplementary Figure 2A–2F). Similarly, ARHGAP39 expression was significantly correlated with age, weight, cancer grade, clinical stage, and TP53 mutation (*p* < 0.001), while patients of the N classification showed no significant difference. In short, we hypothesized that ARHGAP39 expression was linked to clinic pathological features.

### DNA methylation of ARHGAP39

Designed to penetrate the molecular mechanism of ARHGAP39 expression, DNA methylation was investigated. First of all, we assessed the correlation between promoter methylation and ARHGAP39 expression in HCC tissues and para-carcinoma tissues via the UALCAN web resource (Supplementary Figure 3A), which indicated that promoter methylation is more common in carcinoma tissues than in para-carcinoma tissues. Next, the MEXPRESS website was employed to further explore the link between DNA methylation and ARHGAP39 (Supplementary Figure 3B). Generally, 48 CpG sites were linked to ARHGAP39 expression, among which 33 sites were positively connected, accounting for the majority of CpG sites. Subsequently, to explore the position distribution on the chromosomes of the above CpG sites, the SMART website was utilized (Supplementary Figure 3C, 3D). The results showed that the majority of sites, such as cg23111970, cg11574184, cg13514324, cg21421171, cg26006117, cg00063503, cg26861237, and cg26006117, were located on the Island and North Shore. All in all, our results demonstrated ARHGAP39 was hypermethylated in HCC.

### Over-expression of ARHGAP39 predicted poor prognosis of HCC patients

**Figure 2:**
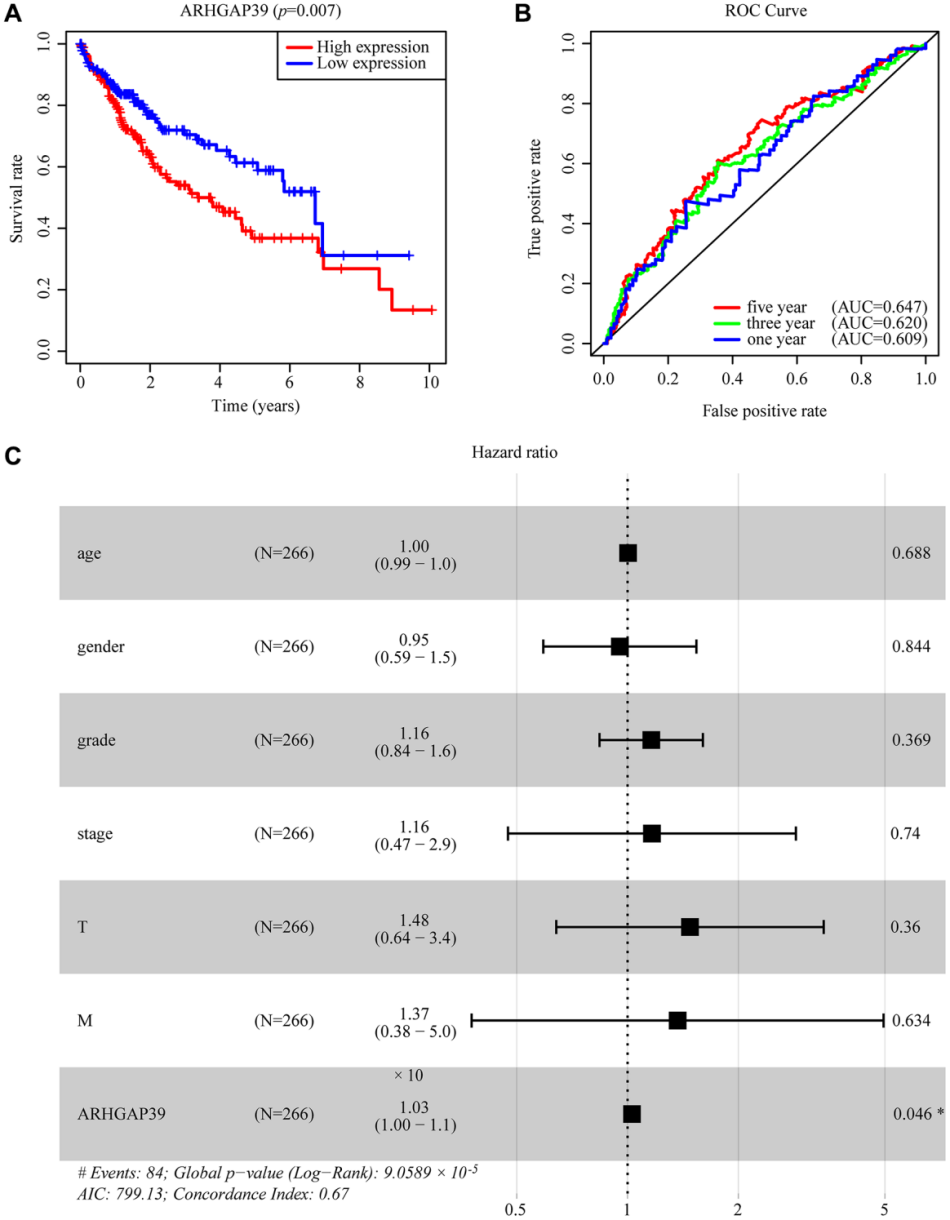
**The effectiveness of ARHGAP39 in predicting prognosis.** (**A**) HCC patients with a higher expression level of ARHGAP39 had an unfavorable prognosis (*p* = 0.007). (**B**) ROC curves for the 1-, 3-, and 5-year survival according to the expression level of ARHGAP39. AUC, area under the curve; ROC, receiver operating characteristic. (**C**) A forest plot of the results of the multivariate analysis. ^*^p < 0.05; ^**^p < 0.01; ^***^p < 0.001. Abbreviations: HR: hazard ratio; CI: confidence interval; T: tumor; N: node, M: metastasis; OS: overall survival; AIC: Akaike’s information criterion.

Aiming at investigating the prognosis of HCC patients when ARHGAP39 was upregulated, a series of approaches were use. Initially, on the basis of the TCGA database, we could obviously find that the high expression of ARHGAP39 tended to an unfavorable prognosis (Figure 2A, *p* < 0.05). What is more, the area under the ROC curve is greater than 0.5, including 1-, 3-, and 5-year incidences of survival of 0.609, 0.620, and 0.647, respectively, which clarifies the fact that up-regulation of ARHGAP39 had an awful prognosis (Figure 2B). On top of that, Kaplan-Meier survival curve analyses were performed, whose results showed that individuals with over-expressed ARHGAP39 had a poorer prognosis across the OS, RFS, PFS, and DSS (Supplementary Figure 4A–4D, *p* < 0.05). Simultaneously, HCCDB databases also produced a consistent result (Supplementary Figure 4E). In addition, we used the UALCAN website to investigate the prognostic variation caused by ARHGAP39 expression differences in the same clinic pathological characteristics. As expected, high expression of ARHGAP39 with the same gender, weight, and grade led to an undesirable prognosis (Supplementary Figure 4F–4H). In addition, univariate and multivariate Cox regression analysis elucidated that ARHGAP39 was an independent prognosis factor for HCC patients (Supplementary Table 2). And the forest plot expressed the equivalent implication (Figure 2C). In conclusion, we found that over-expression of ARHGAP39 predicted an unfavorable prognosis for HCC patients and that ARHGAP39 was an independent prognostic factor.

### ARHGAP39 is associated with cell cycle and metabolic pathways in HCC

**Figure 3:**
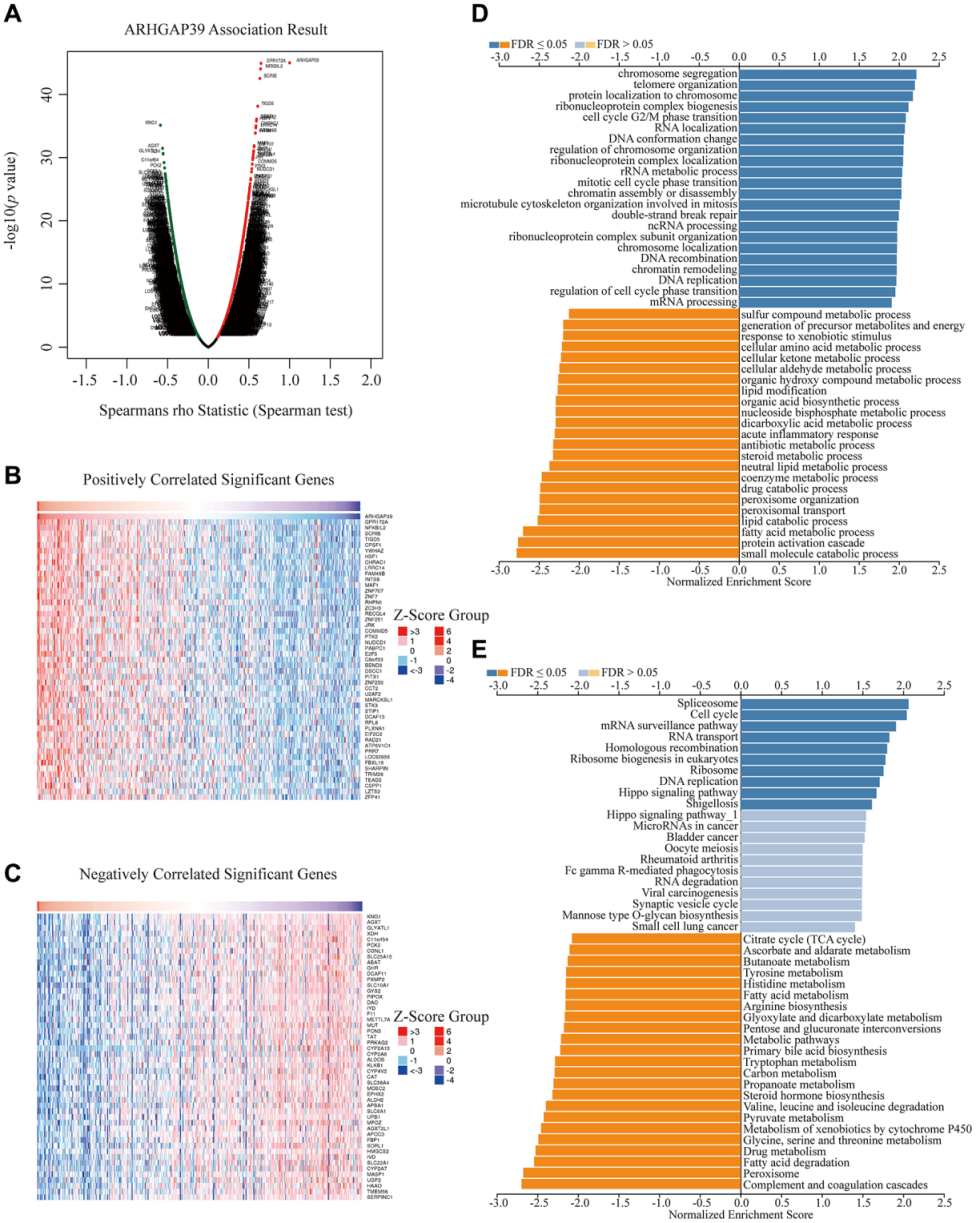
**Enrichment analysis of ARHGAP39 functional networks in HCC.** (**A**) A correlation analysis with spearman's rho value (*p* value 0.05) was used to assess correlations between ARHGAP39 and genes differentially expressed in HCC. (**B**, **C**) Heat maps show genes positively and negatively correlated with ARHGAP39 in HCC (Top 50). (**D**) GO pathway analysis. Dark blue and orange indicate FDR ≤ 0.05, light blue and orange indicate FDR > 0.05 in A. FDR, false discovery rate. (**E**) KEGG pathway analysis. Dark blue and orange indicate FDR ≤ 0.05, light blue and orange indicate FDR > 0.05 in. FDR *q*-val: false discovery rate.

We performed a number of analyses to gain a thorough understanding of ARHGAP39’s biological roles. The LinkOmics website was used to hunt for the co-expression genes, thus discovering a multitude of genes shown in the volcano map (Figure 3A). Next, our group selected the top 100 most relevant genes according to the correlation, among which 50 genes were positively correlated and the other 50 were negatively correlated (Figure 3B, 3C). Subsequently, we performed the GO and KEGG analyses (Figure 3D, 3E). ARHGAP39 was remarkably enriched in several terms as a result of BP, for instance, chromosome segregation, cell cycle G2/M phase transition, mitotic cell cycle phase transition, DNA replication, regulation of cell cycle phase transition, and so on. What’s more, the KEGG analysis results revealed that ARHGAP39 was obviously enriched in the spliceosome, cell cycle, DNA replication, metabolic pathways, pyruvate metabolism, tyrosine metabolism, and so on. Additionally, we took advantage of the LinkOmics website to explore the enrichment pathway in GSEA part. We found that ARHGAP39 was prominently enriched in the cell cycle, spliceosome, hippo signaling pathway, and RNA transport (Supplementary Figure 5A). Overall, our results illustrated that ARHGAP39 was enriched in the cell cycle, spliceosome, and a multitude of metabolic pathways.

### Establish PPI network of co-expression genes

For the purpose of constructing the PPI network of ARHGAP39 as well as tapping into the potential hub genes, the top 500 most related genes in the volcano plot were employed to establish with STRING database (Supplementary Figure 5B). Afterwards, with Cytohubba’s MCC method, the top 10 most relevant proteins in interactive correlation were picked out to find the biological function (Supplementary Figure 5C). They were NOP58, PDCD11, NOP56, FBL, RBM28, BOP1, NIP7, BMS1, DCAF13, and PES1, potentially identified as 10 hub genes. Then, our study probed into their expression in HCC and evaluated their prognostic values, which illustrated that these hub genes were upregulated in HCC patients (Supplementary Figure 6). The 10 hub genes were relevant to the poor OS of HCC patients, while the expression of NOP58, PDCD11, NOP56, FBL, RBM28, BMS1, DCAF13, and PES1 was linked to worse RFS in HCC patients (Supplementary Figure 7). Together, these ten genes may have contributed to the awful prognosis of HCC patients. Furthermore, the HCC meta co-expression network was constructed by the HCCDB database (Supplementary Figure 5D). As expected, we discovered that some genes were involved in cell cycle regulation.

### ARHGAP39 is link to immune infiltration and escape in HCC

**Figure 4:**
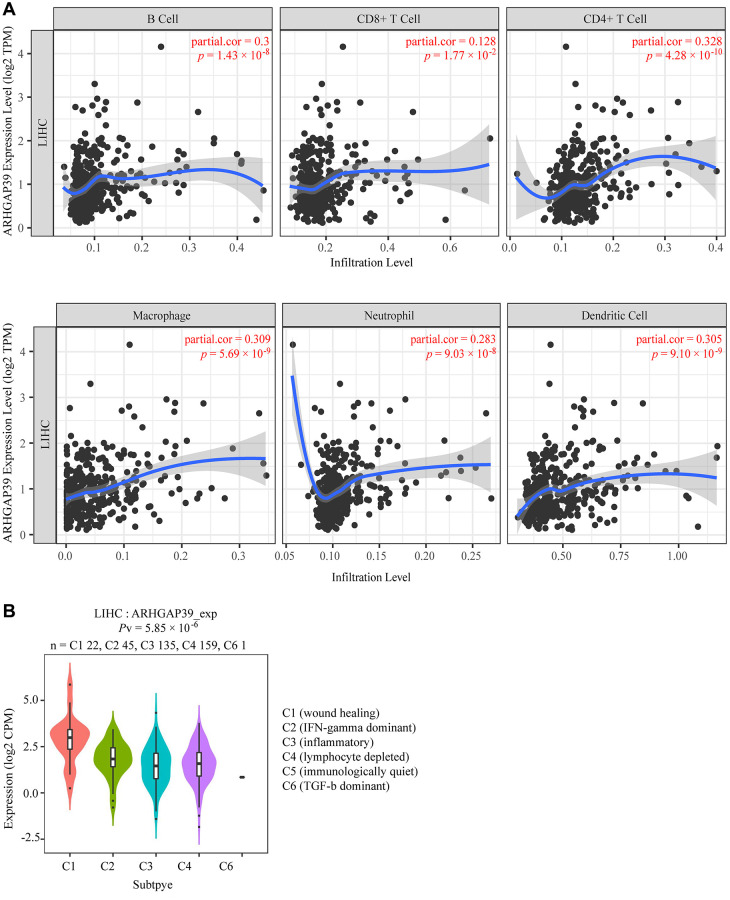
**Correlations of ARHGAP39 expression with immune infiltration level.** (**A**) ARHGAP39 expression is positively related to infiltrating levels of B cells, CD8 + T cells, CD4 + T cells, Macrophage, Neutrophils, and Dendritic Cells in HCC. (**B**) Expression of ARHGAP39 in distinct immune subtypes.

A multitude of advances have shown that tumor immune cell infiltration could affect the efficacies of chemotherapy and immunotherapy and the prognosis of tumor patients. However, the relevance between ARHGAP39 expression and immune infiltration is still unknown in HCC. Therefore, we used TIMER web resource to explore the sealed correlation. The results illustrated that ARHGAP39 expression was positively correlated with immune cell infiltration: B cells (r = 0.3, *p* = 1.43e−08), CD8 + T cells (r = 0.128, *p* =1.77e−02), CD4 + T cells (r = 0.328, *p* = 4.28e−10), macrophages (r = 0.309, *p* = 5.69e−09), neutrophils (r = 0.283, *p* = 9.03e−08), and dendritic cells (r = 0.305, *p* = 9.10e−09), respectively (Figure 4A). ARHGAP39 was found to be highly expressed in the C1 (wound healing) and C2 (IFN- dominated) subgroups, but not in the C6 (TGF- dominant) subgroup (Figure 4B). Further, we penetrated that there were positive correlations between ARHGAP39 and markers of B cells, T (general) cells, monocytes, TAM, M1, M2 macrophages, CD8 + T cells, neutrophils, natural killer cells (Supplementary Figure 8) and dendritic cells on the level of expression (Supplementary Figure 9A) (Supplementary Table 3). We classified T cells and their marker genes in particular, which was consistent with the reported results and showed a positive correlation (Supplementary Table 4). Our study also suggested the correlation between ARHGAP39 expression and the well-known T-cell checkpoint by using the GEPIA and TIMER web resources, discovering that ARHGAP39 expression was positively related to the expression of CCR8, CTLA-4, HAVCR2, PD-1, STAT5B, and TGFB1 (Supplementary Figure 9B–9G). As a consequence, we claimed that ARHGAP39 may be linked to immune infiltration and escape in HCC.

### Prognostic analysis of ARHGAP39 expression based on immune cells in HCC

**Figure 5:**
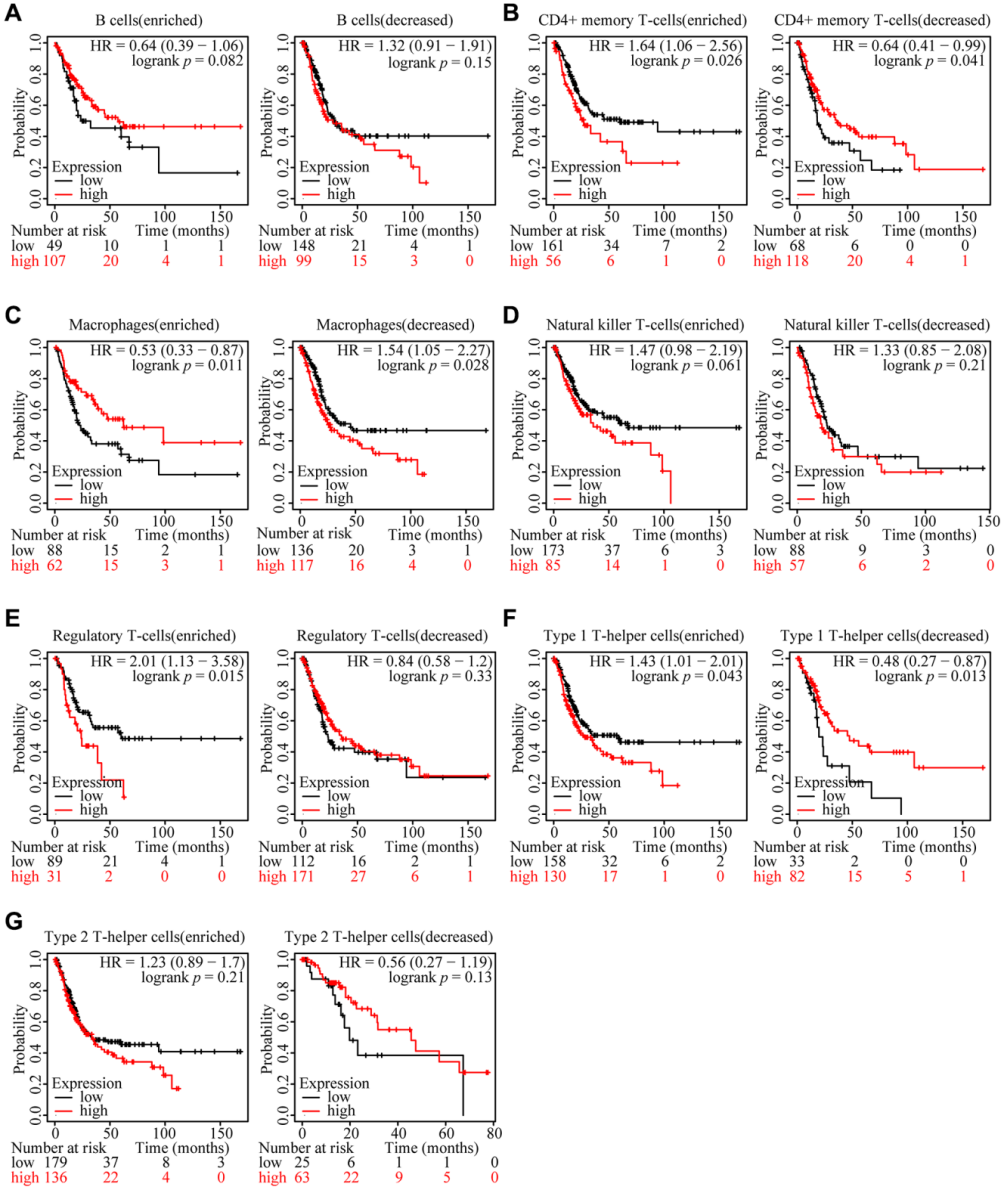
**Relationships between ARHGAP39 of different immune cell subgroups and prognoses in HCC.** (**A**) B cells. (**B**) CD4+ memory T-cells. (**C**) Macrophages. (**D**) Natural killer T-cells. (**E**) Regulatory T-cells. (**F**) Type 1 T-helper cells. (**G**) Type 2 T-helper cells.

**Figure 6:**
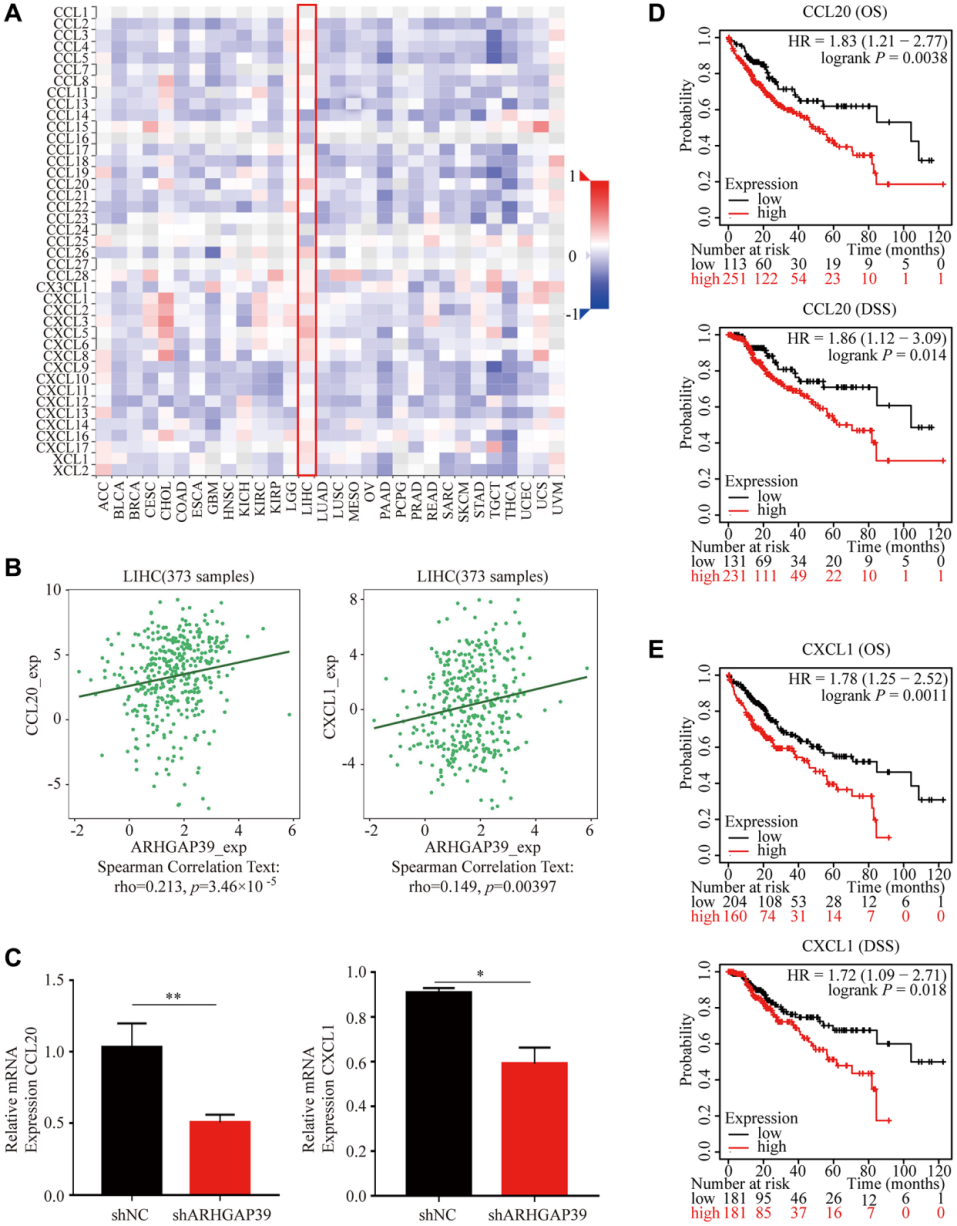
**Correlations between ARHGAP39 expression and HCC-related chemokines.** (**A**) The association between ARHGAP39 and LIHC-related chemokines. (**B**) The scatter diagram showed the correlation between ARHGAP39 and CCL20/CXCL1. (**C**) CCL20/CXCL1 relative mRNA expression in shNC and shARHGAP39. (**D**) OS and DSS of CCL20. (**E**) OS and DSS of CXCL1.

We reasonably hypothesized that the prognosis might be regulated by immune cells based on the external link between ARHGAP39 expression and immune cell infiltration after previously capturing the link between ARHGAP39 expression and poor prognosis of HCC. We first utilized the KM website to analyze the prognosis of HCC patients according to different immune cells and different levels of ARHGAP39 expression (Figure 5A–5G). Notably, the KM survival curve disclosed that patients tended to have an awful prognosis when regulatory T cells were enriched and ARHGAP39 expression levels were high. While regulatory T cells were decreased, even with different levels of ARHGAP39 expression, there was showed no difference in patient survival. However, there were no such differences in the other cells. Therefore, it was suggested that ARHGAP39 may affect patient survival by enriching regulatory T cells. Intriguingly, the phenomenon triggered our thorough exploration of its potential mechanisms. Previous studies have reported that cytokines are the main regulators of the immune system, enabling immune cells to communicate within short distances [37]. With the internal mechanism between chemokines and their receptors, distinct immune cell subtypes are recruited into the tumor micro-environment, contributing to different impacts on cancer progression [38]. By using the TISIDB database, we found some chemokines associated with LIHC, among which eight chemokines were positively correlated (Figure 6A). Then, we selected two of them to explore, namely, CCL20 and CXCL1. Concrete correlations were displayed by the scatter diagram (Figure 6B). Further, we conducted a verification experiment to screen the matched chemokine, exploring the expression of immune-related chemokines in ARHGAP39 knockdown HCC cells. Compared with shNC, the expression of CCL20 and CXCL1 was decreased in ARHGAP39 knockdown cells (Figure 6C). Simultaneously, the up-regulated CCL20 and CXCL1 predicted an undesirable prognosis (Figure 6D, 6E). Furthermore, we predicted patient response rates to immunotherapy with different expressions of ARHGAP39, and we found the group with high ARHGAP39 expression had higher TIDE score, which means poor efficacy of immune checkpoint blocking therapy (ICB) and short survival after ICB treatment (Supplementary Figure 10). It was reasonable to reach the conclusion that ARHGAP39 expression was associated with poor prognosis through chemokines recruiting regulatory T cells, thus contributing to their increased levels of infiltration.

### The association between ARHGAP39 expression and m^6^A modification in HCC

**Figure 7:**
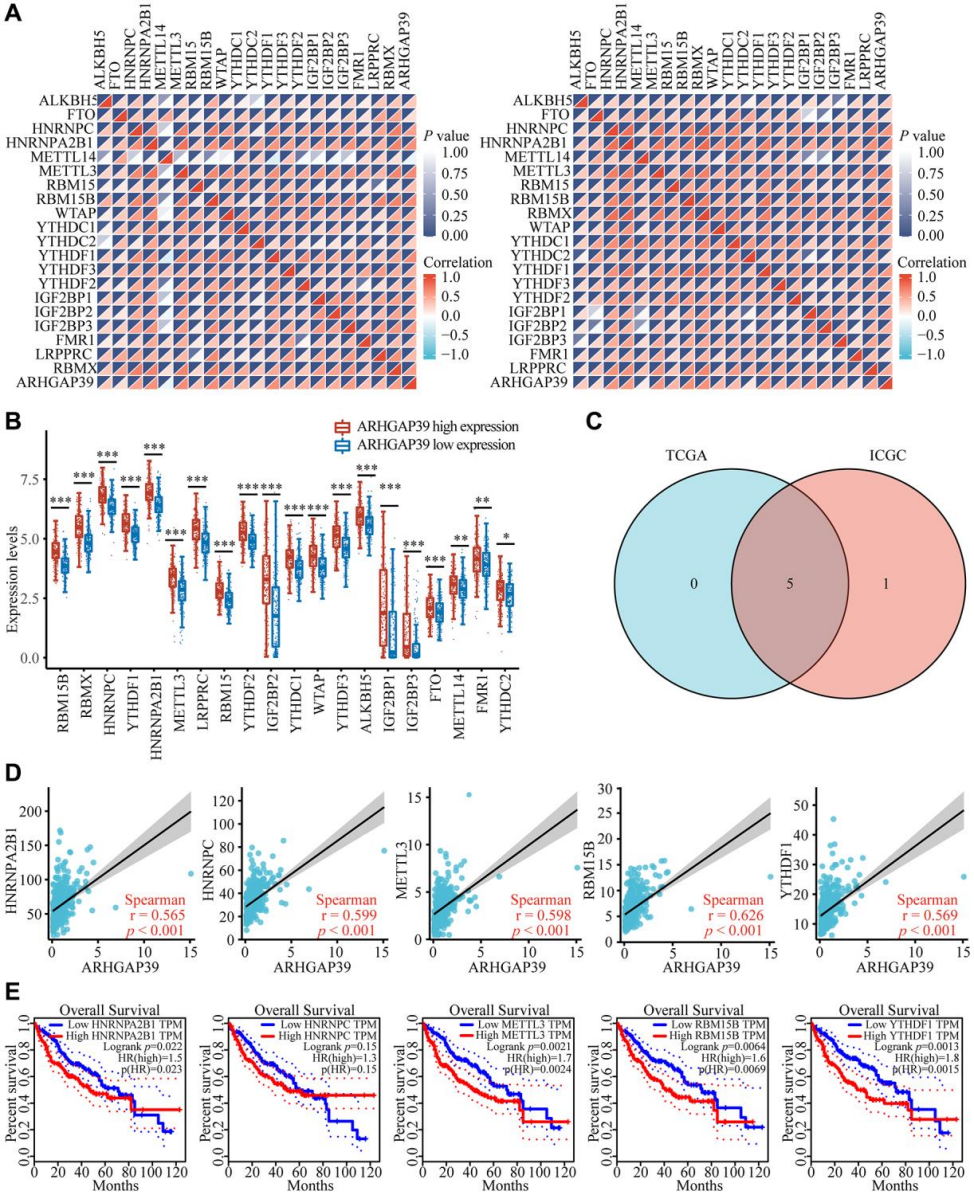
**Corrections of ARHGAP39 expression with m^6^A modification in HCC.** (**A**) The correlation between ARHGAP39 expression and the expression of m^6^A modified genes was investigated by the Spearman statistical method using the TCGA and ICGC databases. (**B**) Distinct m^6^A-related gene expression in HCC patients with different expressions of ARHGAP39. (**C**) Five genes were found in the intersection between the TCGA and ICGC databases. (**D**) The correlation between ARHGAP39 and m^6^A modified genes were analyzed by the scatter plot. (**E**) The overall survival of HCC patients was separated into two groups of high and low expression of these five m^6^A related genes. ^*^p < 0.05; ^**^p < 0.01; ^***^p < 0.001.

Growing evidence has shown that N6-methyladenosine (m^6^A) RNA modification plays a significant part in cancer biology [39]. The regulators of m^6^A play multiple roles in cancer development, for instance, proliferation, migration, and invasion [40]. To explore whether there was a correlation between ARHGAP39 and m^6^A modification, we applied the TCGA and ICGC databases to seek the association between ARHGAP39 expression and 20 m^6^A connected genes in HCC. ARHGAP39 expression was positively correlated with many m^6^A related genes (Figure 7A). To determine whether there were differences in expression of m^6^A relevant genes between the different expression groups of ARHGAP39 in LIHC, we used the TCGA database (Figure 7B), which revealed that m^6^A associated gene expression was higher in the ARHGAP39 high expression group compared to the low expression group. Afterwards, we screened five m^6^A genes with a genetic correlation greater than 0.5 using the Venn plot with the TCGA and ICGC databases (Figure 7C). They were, respectively, HNRNPA2B1, HNRNPC, METTL3, RBM15B, and YTHDF1. The scatter gram demonstrated their specific relationships (Figure 7D). Further studies have shown that over-expression of HNRNPA2B1, METTL3, RBM15B, and YTHDF1 predicted a bad prognosis in HCC patients (Figure 7E). So, our results suggested that ARHGAP39 may affect HCC through the m^6^A related genes.

### ARHGAP39 protein interaction network and molecular docking model

**Figure 8:**
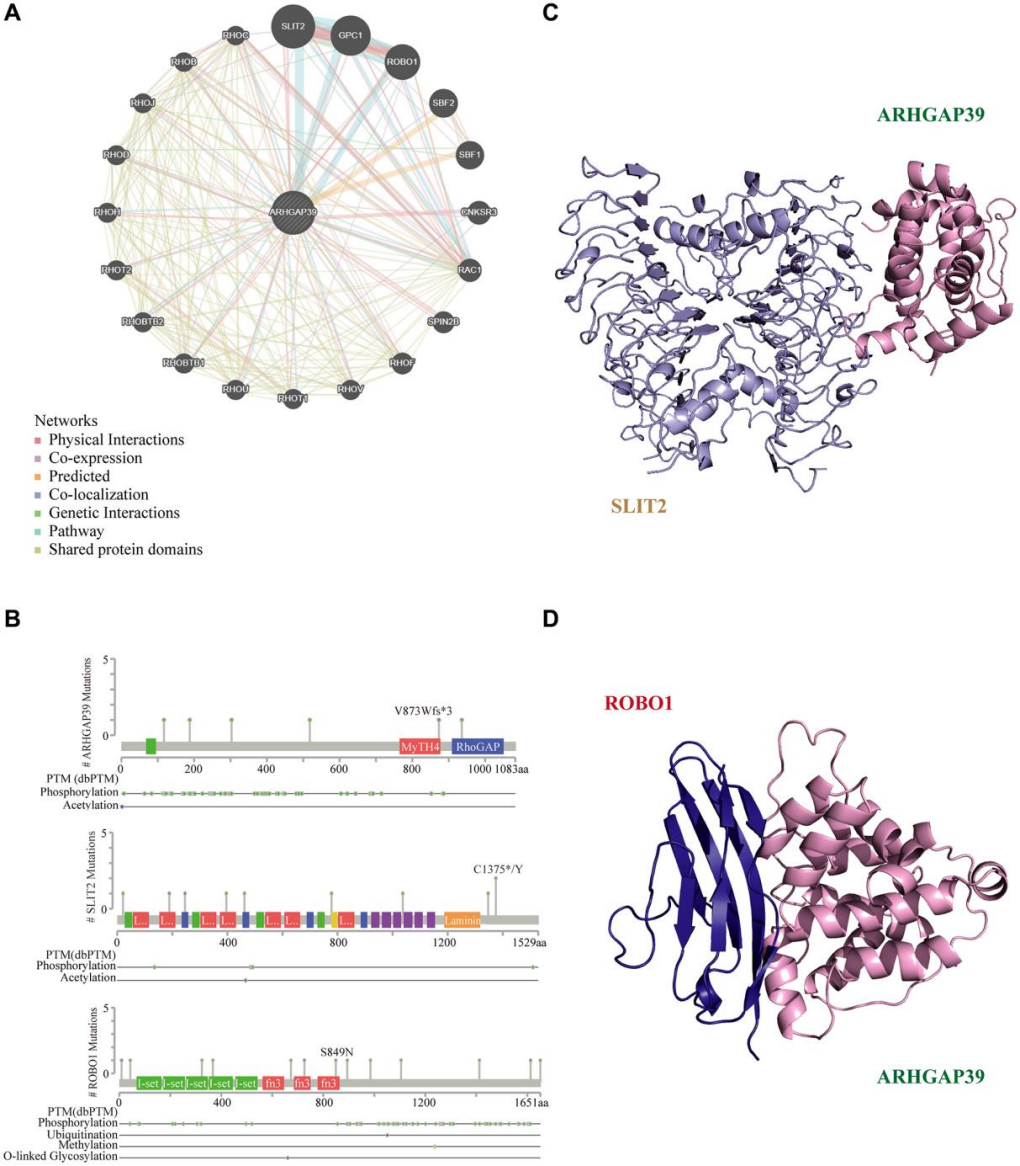
**Molecular docking analysis.** (**A**) ARHGAP39 interconnection network built by GeneMANIA; (**B**) Protein secondary structure of ARHGAP39, SLIT2, and ROBO1. (**C**) The structure of ARHGAP39 and SLIT2 combined from the perspective of cartoon. (**D**) The structure of ARHGAP39 and ROBO1 combined from the perspective of cartoon.

Proteins usually serve as team members in a dynamic network. Increasing advances indicate that PPI is vital in massive biological processes in cells [41]. Therefore, the GeneMANIA database was utilized to build an interaction network between ARHGAP39 and other cancer-related proteins (Figure 8A). The result indicated that ARHGAP39 physically interacted with 18 proteins. Notably, we detected an apparent protein-protein interaction among ARHGAP39, SLIT2, and ROBO1. Next, we hunted for the secondary structures of them with the cBioPortal database (Figure 8B), among which were different chemical modification sites, such as phosphorylation, acetylation, ubiquitination, methylation, and O-linked glycosylation. Additionally, using the SWISS-MODEL and PDB databases, we predicted the tertiary structures of ARHGAP39, SLIT2, and ROBO1. Further, SLIT2 and ROBO1 were reported to promote the migration of hepatocellular carcinoma cells. On account of the significance of SLIT2 and ROBO1 in HCC, we forecast the potential binding domain among ARHGAP39, SLIT2, and ROBO1 via the ZDOCK server (Figure 8C, 8D).

### Cancer pathway and interacting chemicals analysis of ARHGAP39

**Figure 9:**
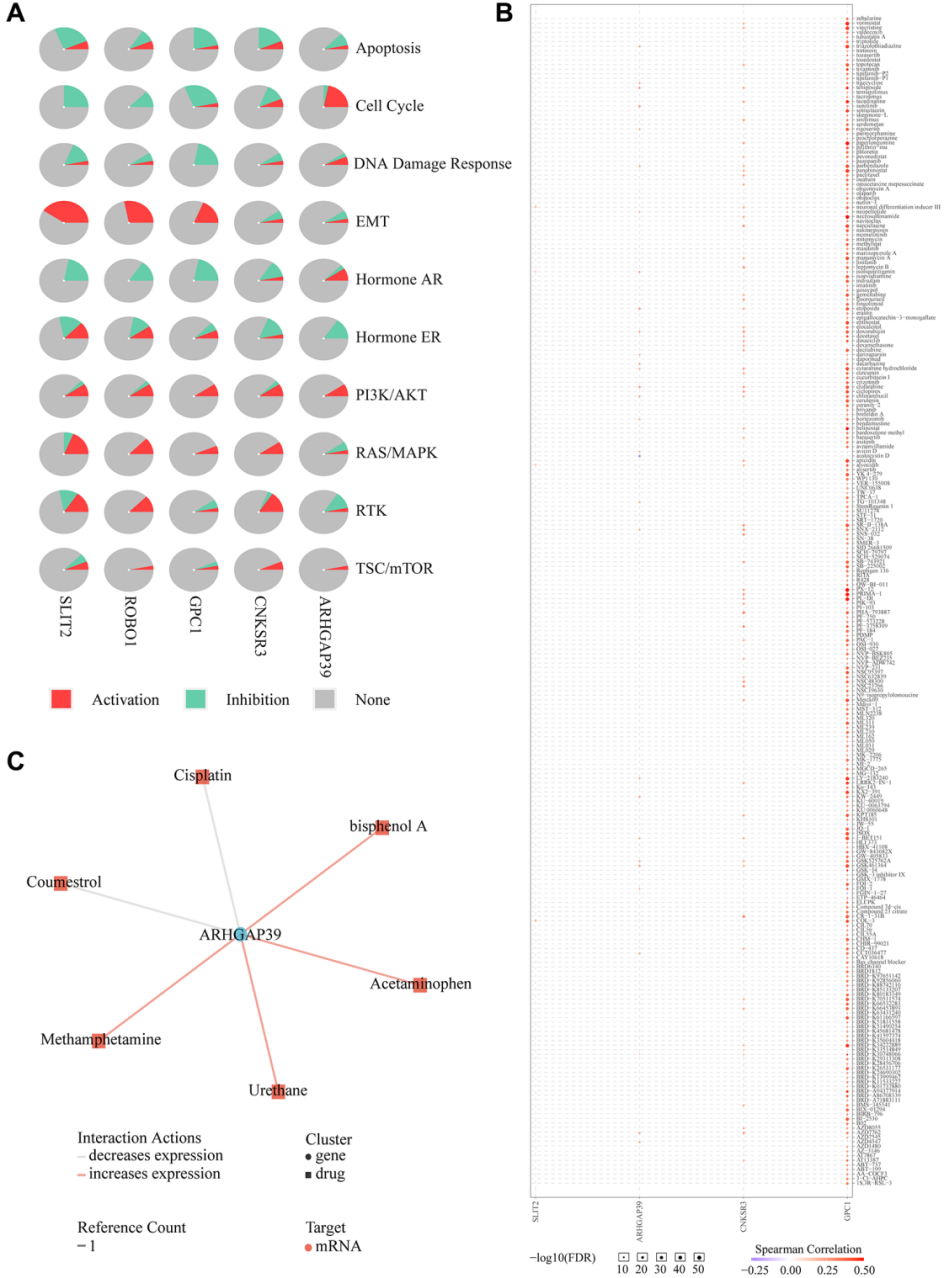
**Drug susceptibility analysis associated with ARHGAP39.** (**A**) Pathway analyses were studied by GSCA Lite website. (**B**) We used the GSCA Lite website to display drug susceptibility with five genes (**C**) Interacting chemicals of ARHGAP39 from CTD.

Drug resistance takes up a large proportion of the undesirable influences of chemotherapy in HCC [42]. We first chose four physically interacted genes from the GeneMANIA website to detect the cancer pathway in HCC patients on the GSCA website. They are SLT2, GPC1, ROBO1, and CNKSR3, shown in Figure 8A. Further pathway analysis revealed that over-expression of ARHGAP39 significantly activates the cell cycle (Figure 9A). Furthermore, patients with high ARHGAP39 expression can activate the DNA Damage Response, hormone AR, PI3K/AKT, and TSC/mTOR pathways, as well as inhibit apoptosis, hormone ER, and EMT pathways. Besides, the EMT pathway was significantly activated by GPC1, ROBO1, and SLIT2. The drug sensitivity analysis was performed by the GSCA website to find the sealed interactions between ARHGAP39 and cancer chemotherapeutic drugs, and the results showed that cells with high expression of ARHGAP39 are resistant to 28 drugs, such as sunitinib, etoposide, clofarabine, and so on, and sensitive to 1 drug, namely austocystin D (Figure 9B). Furthermore, the CTD database constructed a chemotherapeutics drug-gene interaction network, indicating that 6 drugs could influence the expression of ARHGAP39, with coumestrol and cisplatin being the two drugs that could decrease its expression level (Figure 9C). In a nutshell, our discovery disclosed that ARHGAP39 activated the cell cycle and illustrated the drug interactions in patients, making it conducive to assisting the therapy of HCC patients to a certain extent.

## DISCUSSION

HCC accounts for more than 90% of primary liver tumors [43]. Simultaneously, its morbidity and mortality are high, with 841,080 cases diagnosed in 2018 and an estimated 1,361,836 cases in 2040, and a five-year survival rate of only 18%. As is well known, AFP has been widely used as a biomarker for the diagnosis of hepatic malignant tumors as a typical example, but screening still has great limitations due to its low sensitivity [44]. Because of this, it is urgent to identify more effective biomarkers for detection, prognostic evaluation, and treatment options for HCC patients. This study indicated that ARHGAP39 appears to be a promising candidate prognostic factor and a target for therapy.

We first noticed that several online websites showed that ARHGAP39 was abnormally highly expressed in hepatocellular carcinoma tissues, which was the same as the analysis conclusions of the TCGA and ICGC databases. Furthermore, ARHGAP39 expression was related to clinicopathological features such as age, grade, stage, and T classification. Subsequently, the survival curve suggested that patients with over-expression of ARHGAP39 generally had a poor survival rate, not only in OS but also in DSS, PFS, and RFS. ROC curve and Cox regression analyses also illustrated the prognostic value of ARHGAP39 as an independent prognostic factor.

In GO results, ARHGAP39 has a strong connection with the biogenesis of chromosome segregation, cell cycle G2/M phase transition, mitotic cell cycle phase transition, DNA replication, and regulation of cell cycle phase transition. Besides, KEGG analysis confirmed that ARHGAP39 was particularly enriched in the spliceosome, cell cycle, DNA replication, metabolic pathways, pyruvate metabolism, and tyrosine metabolism. We know that not only the cell cycle [45], but also metabolic pathways, play a role in the progression of hepatocellular carcinoma [46, 47]. For example, reduced tyrosine metabolism activates the cell cycle and promotes cell proliferation [48], and enzymes involved in glycolysis, such as Pyruvate kinase-Pyruvate kinase M2 (PKM2), are highly expressed and are strongly related to a bad OS in HCC [46]. Besides, the LinkOmics dataset identified the key roles of ARHGAP39 in the cell cycle, hippo signaling pathway, spliceosome, and RNA transport. Furthermore, the results of pathway analysis using the GSCA website also indicated that the over-expression of ARHGAP39 could activate the cell cycle pathway. In conclusion, these results indicate that ARHGAP39 may promote the tumorigenesis and progression of hepatocellular carcinoma by participating in cell cycle and metabolism related pathways.

The cell cycle directs mitosis through a number of regulatory proteins that eventually give rise to two daughter cells [49]. There is no doubt that genes play different roles in the cell cycle. For instance, Ago2 is involved in the cell cycle in prostate cancer (PCA) [50, 51]; and as a chromosomal passenger complex (CPC), RCC2 plays a non-negligible role in all cell cycle phases [52]; JRK is related to a chromosomal centromeric locus in G(2) [53]. We applied the HCCDB website to explore the co-expressed genes of ARHGAP39, and it was found that ARHGAP39 was closely related to Ago2, RCC2, and JRK proteins, which again confirmed that ARHGAP39 may have a significant influence on the regulation of the cell cycle progression.

HCC is a malignant tumor associated with inflammation, and its immune micro-environment can establish a symbiotic relationship with tumor cells [54]. The tumor micro-environment (TME) contains multiple types of immune cells [55], and some studies have confirmed that changes in the number and function of immune cells may be beneficial for HCC [56]. Our study illustrates that the expression of ARHGAP39 showed the same trend as the infiltration level of various immune cells, including B cells, CD8+T cells, CD4+ cells, macrophages, neutrophils, and dendritic cells. Meanwhile, ARHGAP39 expression was positively related to the expression of a variety of immune cell markers, especially B cells, T cells, CD8+ T cells, monocytes, TAM, M1, and DC. The above results confirm our hypothesis that ARHGAP39 expression in HCC is connected with immune cell infiltration.

In addition, chemokines have an indispensable function in the recruitment and localization of immune cells in the TME [57]. At the same time, they can directly target tumor cells and stromal cells, thereby directly and indirectly affecting tumor immunity and influencing cancer progression, tumor treatment, and patient prognosis [58]. Studies have shown that CCL20 accelerates tumor metastasis by inducing epithelial-mesenchymal transformation (EMT), and inhibiting T cell proliferation, and promoting the amplification of immunosuppressive Treg cells [59]. It has also been documented that CXCL1 plays a role in Tregs recruitment and accumulation and promotes angiogenesis in some cancers [60]. Interestingly, in our study, there was a positive association between ARHGAP39 and CCL20 and CXCL1 at the expression level. Coincidentally, the difference in survival caused by the expression of ARHGAP39 in patients only occurred when regulatory T cells were enriched, and we found that high expression of CCL20 and CXCL1 could lead to a poor prognosis for patients. In conclusion, ARHGAP39 may promote the accumulation of Tregs through CCL20 and CXCL1, thus affecting the prognosis of patients.

In other respects, the inhibitory checkpoint, including CTLA-4, PD-1, and PD-L1, which are programmed to transmit inhibitory signals, modulates the balance between T-cell activation, tolerance, and immunopathology to suppress antitumor immune responses in solid tumors [61, 62]. In recent years, immune checkpoint blocking using anti-CTLA-4 and anti-PD-1 antibodies has been successfully applied to tackle some types of advanced tumors, including non-small cell lung cancer (NSCLC), melanoma, bladder cancer, and Hodgkin’s lymphoma. Previously, several studies have also suggested that immunotherapy may provide more possibilities for the cure of HCC [63]. The research revealed that ARHGAP39 expression was positively related to CTLA-4, PDCD1, CCR8, HAVCR2, TGFB1, and STAT5B, bringing new hope for the precise treatment of HCC with immune checkpoint inhibitors (ICI).

N6-methyladenosine (m^6^A) is the most abundant mRNA modification, and m^6^A is involved in almost all steps of RNA metabolism [64]. There is increasing evidence that the m^6^A modification has a significant impact on cancers, including HCC, through various mechanisms [65]. It has been reported that METTL3, YTHDF1, HNRNPA2B1, HNRNPC, and RBM15B are overexpressed in HCC, and most of them can lead to a poor prognosis [66–69], which is similar to our results. In addition, METTL3 promotes the process of HCC by regulating the m^6^A levels of USP7 [70]; the increased expression of YTHDF1 enhances the proliferation of HCC cells, which can be achieved by the connection of circMAP2K4 with HSA-Mir-139-5p [71]. Coincidentally, the ARHGAP39 we studied has a strong positive correlation with METTL3, YTHDF1, HNRNPA2B1, HNRNPC, and RBM15B, both in the TCGA and ICGC databases. This implies that ARHGAP39 may be involved in the m^6^A modification process and consequently affect the progression of HCC.

Subsequently, we also explored the interaction network of ARHGAP39, and the results showed that ARHGAP39 could directly interact with SLIT2 and ROBO1. Some research has demonstrated that ROBO1 can be associated with cell migration through the process of GTPase activity or molecular guided cue response [71]. When ROBO1 is silenced, HCC cell proliferation, migration, invasion, tumor progression, and metastasis are confined [72]. Recently, the role of SLIT2 together with the ROBO1 receptor in tumor growth and metastasis has been explored. The SLIT2/ROBO1 pathway has been shown to be involved in the progression of intrahepatic cholangiocarcinoma (ICC) [73], breast [74] and bowel cancer [75]. Moreover, previous studies have confirmed that SLIT2 knockdown can induce the over-expression of ROBO1 in hepatocellular carcinoma. And both down-regulation of SLIT2 expression and over-expression of ROBO1 can promote tumor growth and metastasis [76]. Protein-protein interaction is the cornerstone of many biological functions. We applied a molecular docking model to predict the binding of ARHGAP39 to SLIT2 and ROBO1, which suggests a new direction for the development mechanism of HCC.

Drug therapy is a crucial means for improving the quality of life and prognosis of HCC patients, but only a few drugs, such as sorafenib, are considered effective methods for the treatment of HCC [77], and drug resistance is an important obstacle to the curative treatment of HCC patients [78]. We found that ARHGAP39 expression was linked to drug sensitivity in HCC patients; that is, patients with ARHGAP39 overexpression were resistant to 28 drugs, particularly sunitinib, etoposide, and clofarabine, but sensitive to austocystin D. It has to be said that the influence of ARHGAP39 in the therapy of HCC patients, which is closely correlated with the resistance of HCC patients to therapeutic drugs, is worthy of further exploration.

In conclusion, based on the bioinformatics analysis method, we identified the important value of ARHGAP39 for prognosis assessment of hepatocellular carcinoma patients and, for the first time, elucidated the possible involvement of ARHGAP39 in important biological processes and functions such as cell cycle, immune infiltration, m^6^A modification, and drug resistance, providing a potential biomarker for diagnosis and prognosis of HCC patients. Meanwhile, ARHGAP39 is also an immunotherapeutic target worthy of deeper exploration.

## Supplementary Materials

Supplementary Figures

Supplementary Tables
