# Supplementary material for: Biological function analysis of ARHGAP39 as an independent prognostic biomarker in hepatocellular carcinoma
Source: Aging (Albany NY). 2023 Apr 5;15(7):2631–66. doi: 10.18632/aging.204635 (PMC10120899; doi:10.18632/aging.204635)
Supplement: Supplementary Figures [file aging-15-204635-s001.pdf]

SUPPLEMENTARY FIGURES

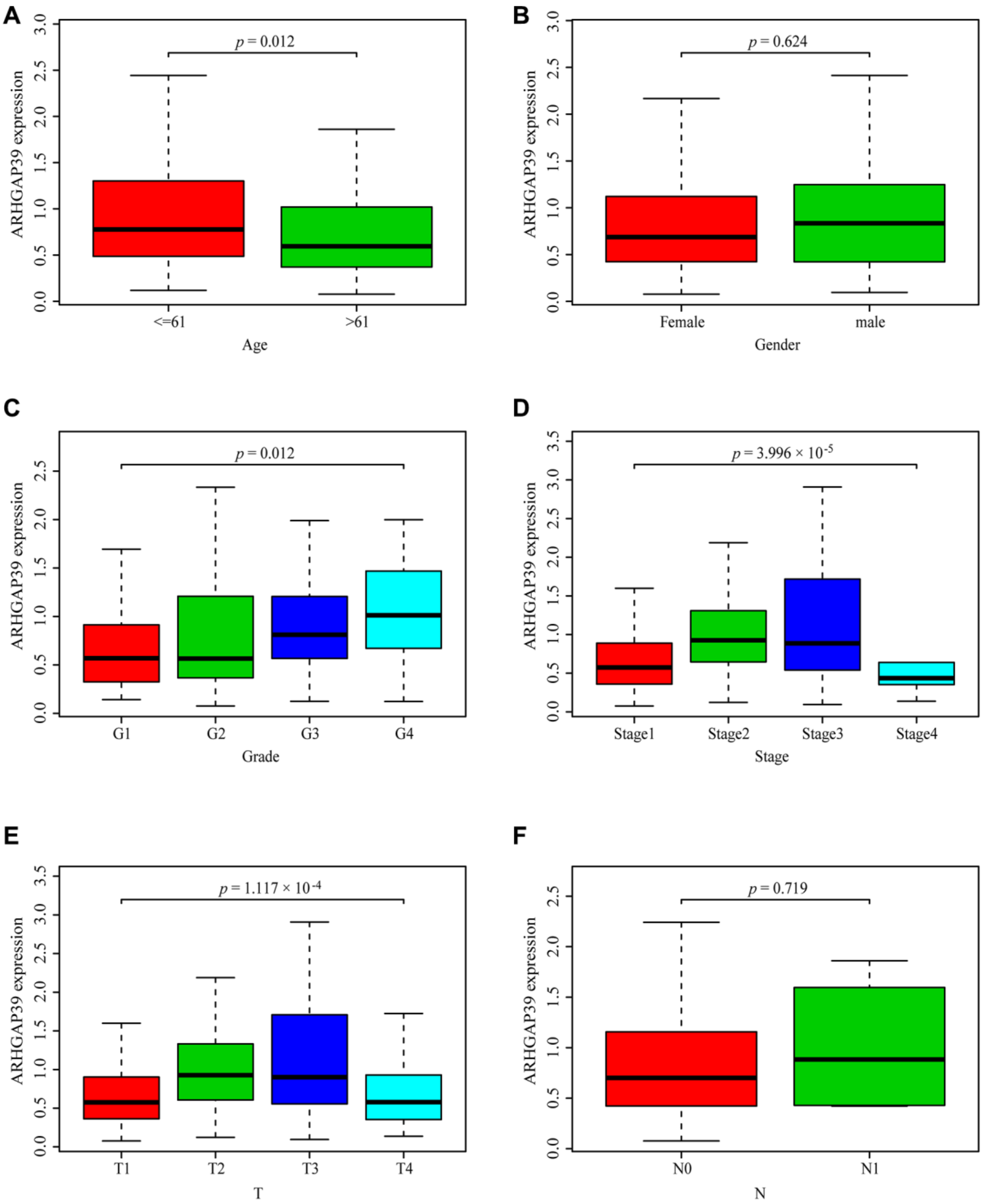

Supplementary Figure 1. Box plots exploring the relationship between ARHGAP39 expression and clinicopathological characteristics. (**A**) Age; (**B**) Gender; (**C**) Grade; (**D**) Stage; (**E**) T; (**F**) N.

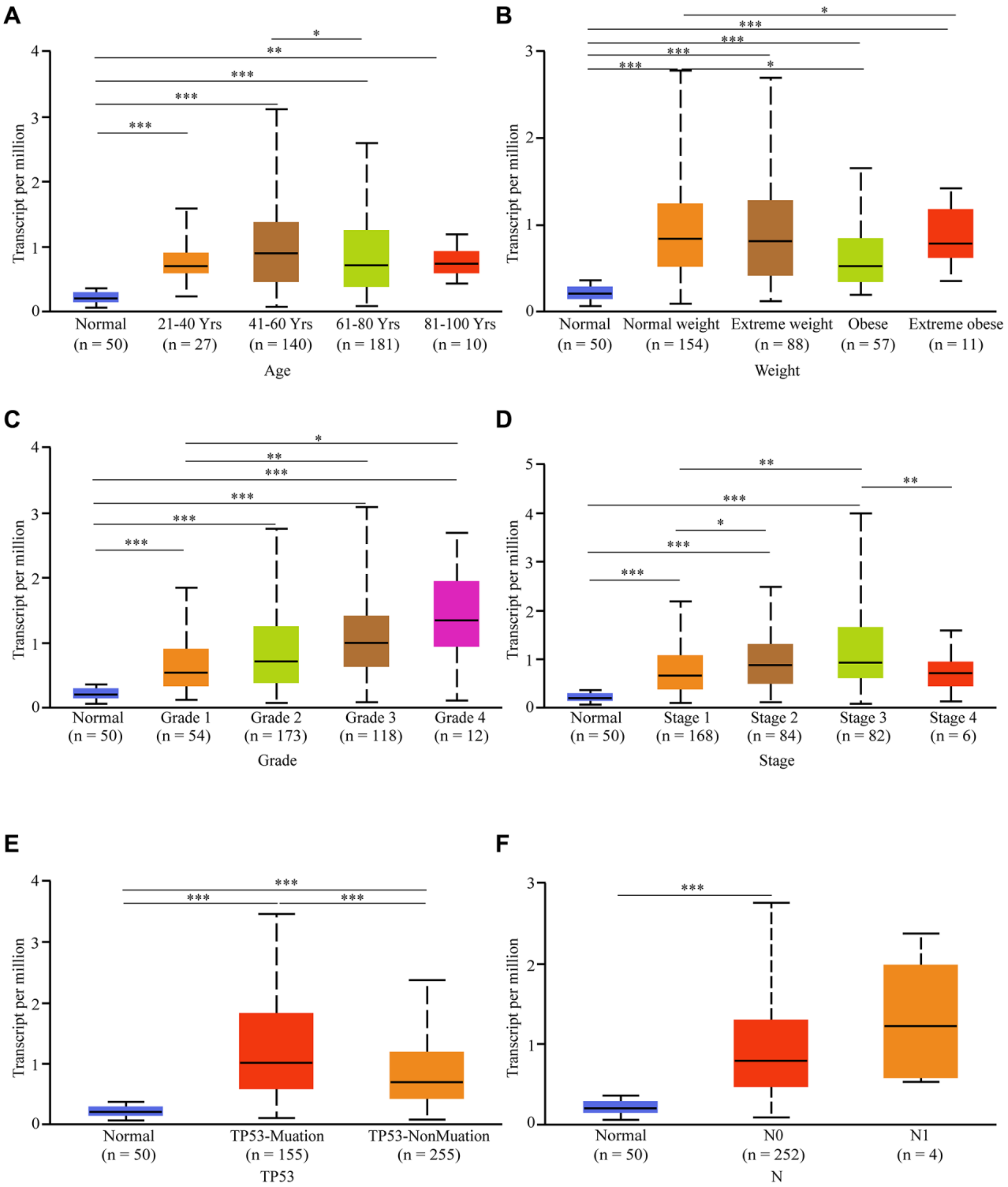

Supplementary Figure 2. The correlation between ARHGAP39 expression and clinicopathological characteristics (**A**) Age; (**B**) weight; (**C**) Grade; (**D**) Stage; (**E**) TP53 Mutation; (**F**) N.

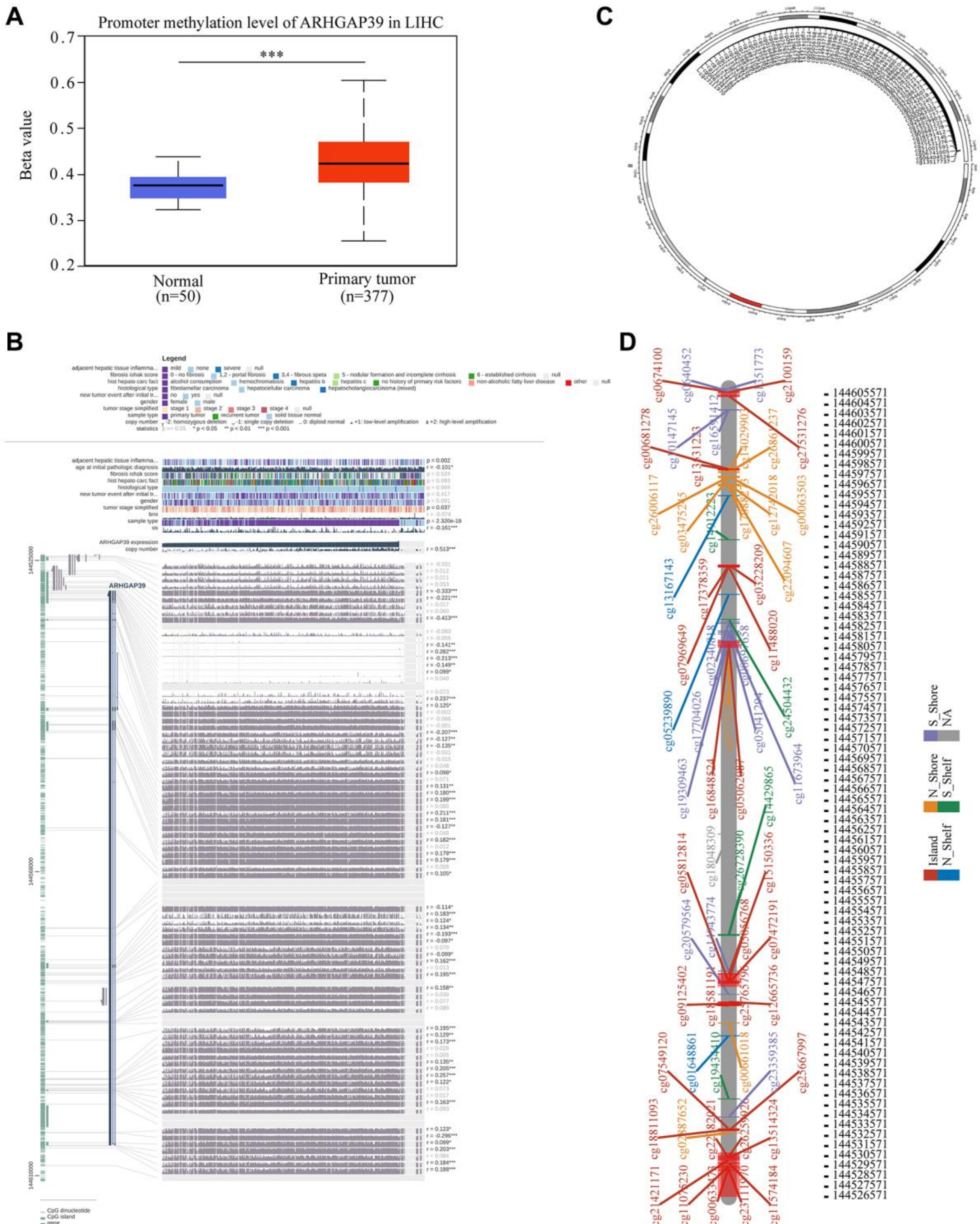

Supplementary Figure 3. DNA methylation of ARHGAP39 in HCC. (**A**) Boxplot comparing the methylation levels of ARHGAP39's promoter in normal and HCC samples. (**B**) The correlation between methylation sites and ARHGAP39 expression. The positional distribution of methylation sites on the chromosomes. (**C**) Overall (**D**) Concretely. $^{*}p < 0.05$; $^{**}p < 0.01$; $^{***}p < 0.001$.

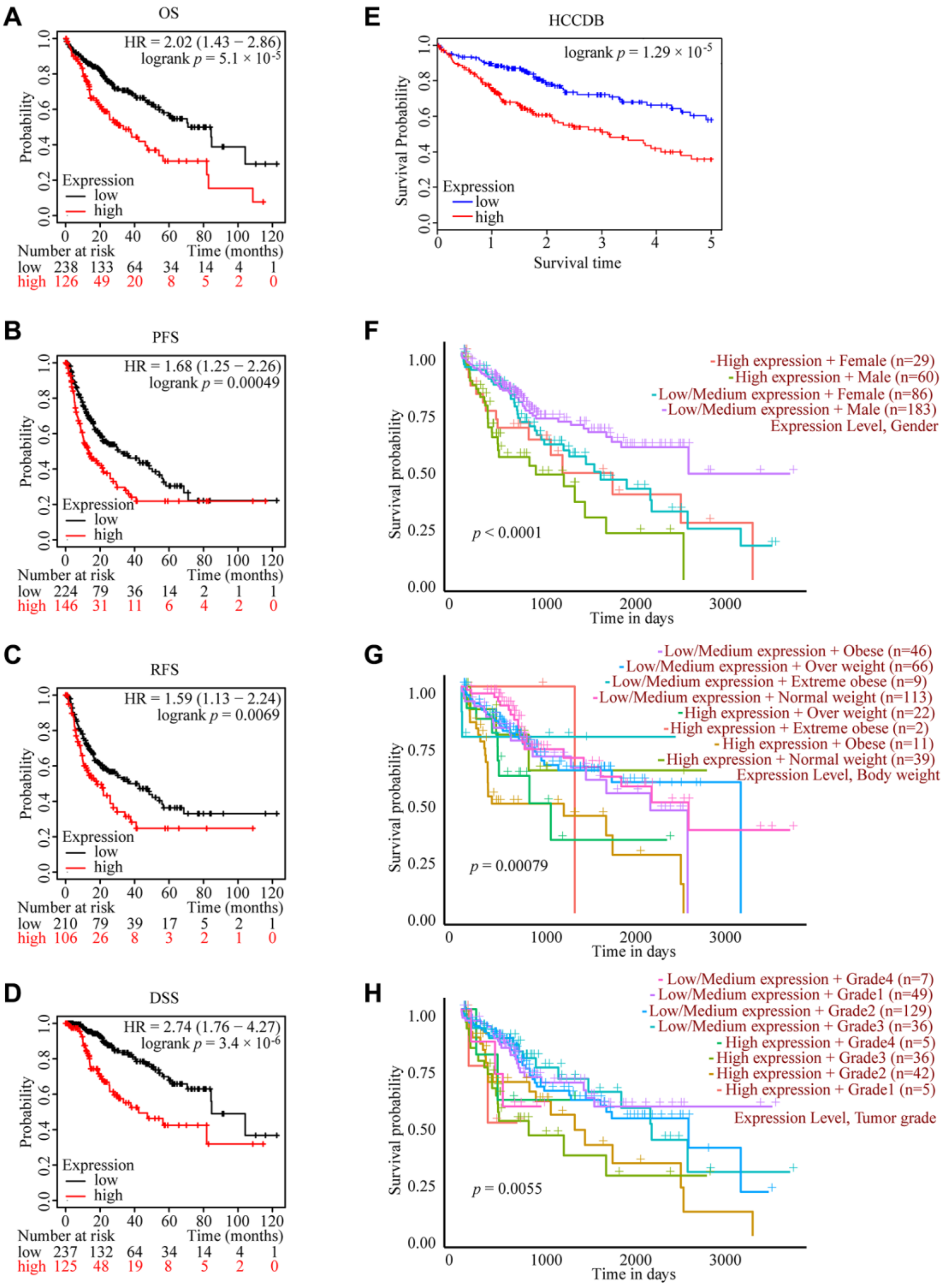

Supplementary Figure 4. The function of high ARHGAP39 expression in prognosis. (**A**–**D**) Kaplan-Meier analysis of OS (overall survival), PFS (progression-free survival), RFS (relapse-free survival), and DSS (disease-specific survival) in HCC patients. (**E**) Effect of ARHGAP39 mRNA expression level on HCC patient survival by HCCDB (p = 1.29e−05). (**F**–**H**) Survival probability of HCC patients with different ARHGAP39 expression and gender, weight, and tumor grade.

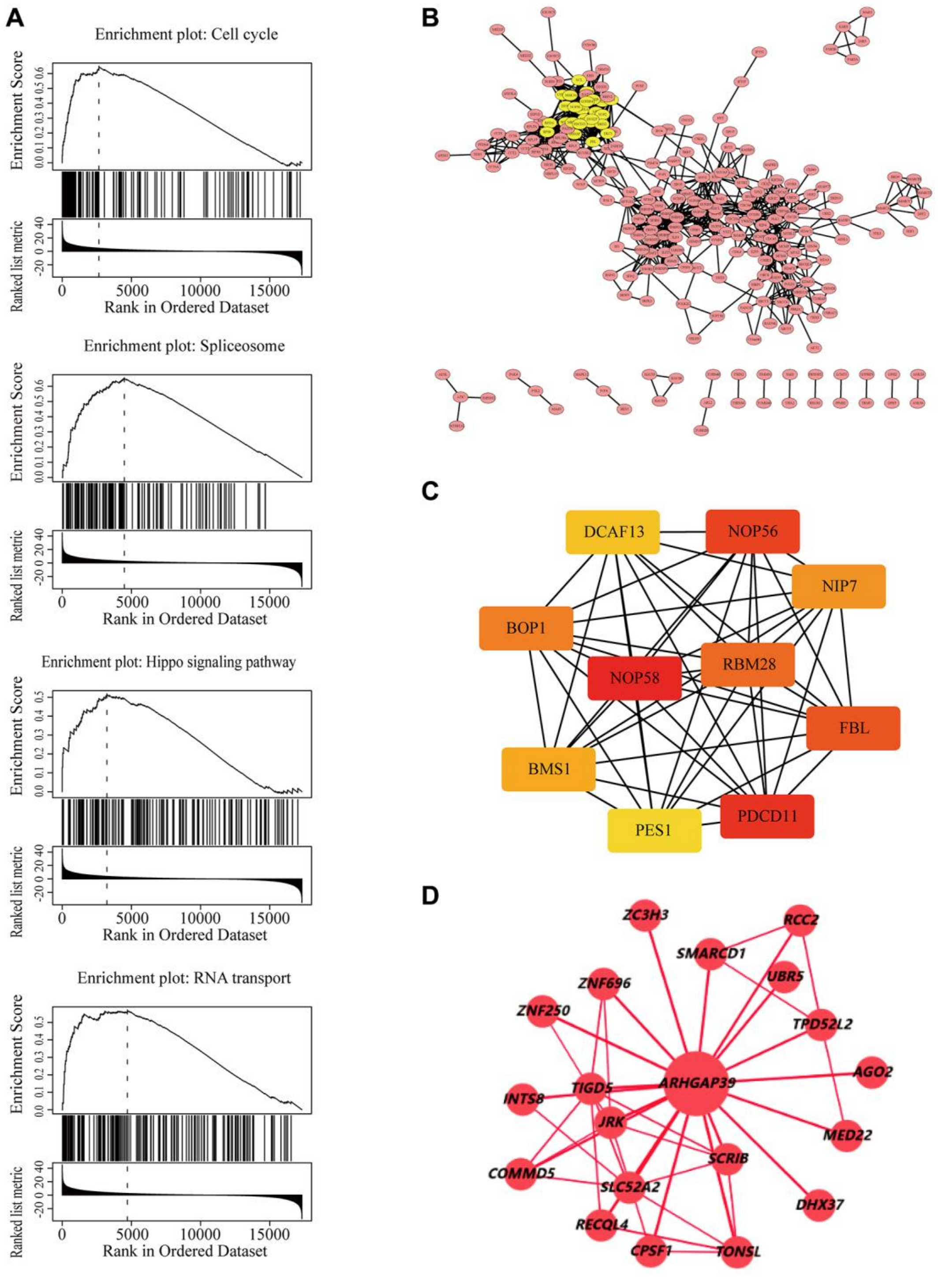

Supplementary Figure 5. Co-expression genes and protein-protein interaction (PPI) network of ARHGAP39 in HCC. (**A**) GSEA analysis of ARHGAP39 based on expression in the TCGA-LIHC dataset. (**B**) The most significant module selected by the MCODE plugin (degree cut-off = 2, node score cut-off = 0.2, k-core = 2, and max. depth = 100). (**C**) The top 10 proteins by Cytohubba's MCC method. (**D**) HCC meta co-expression network.

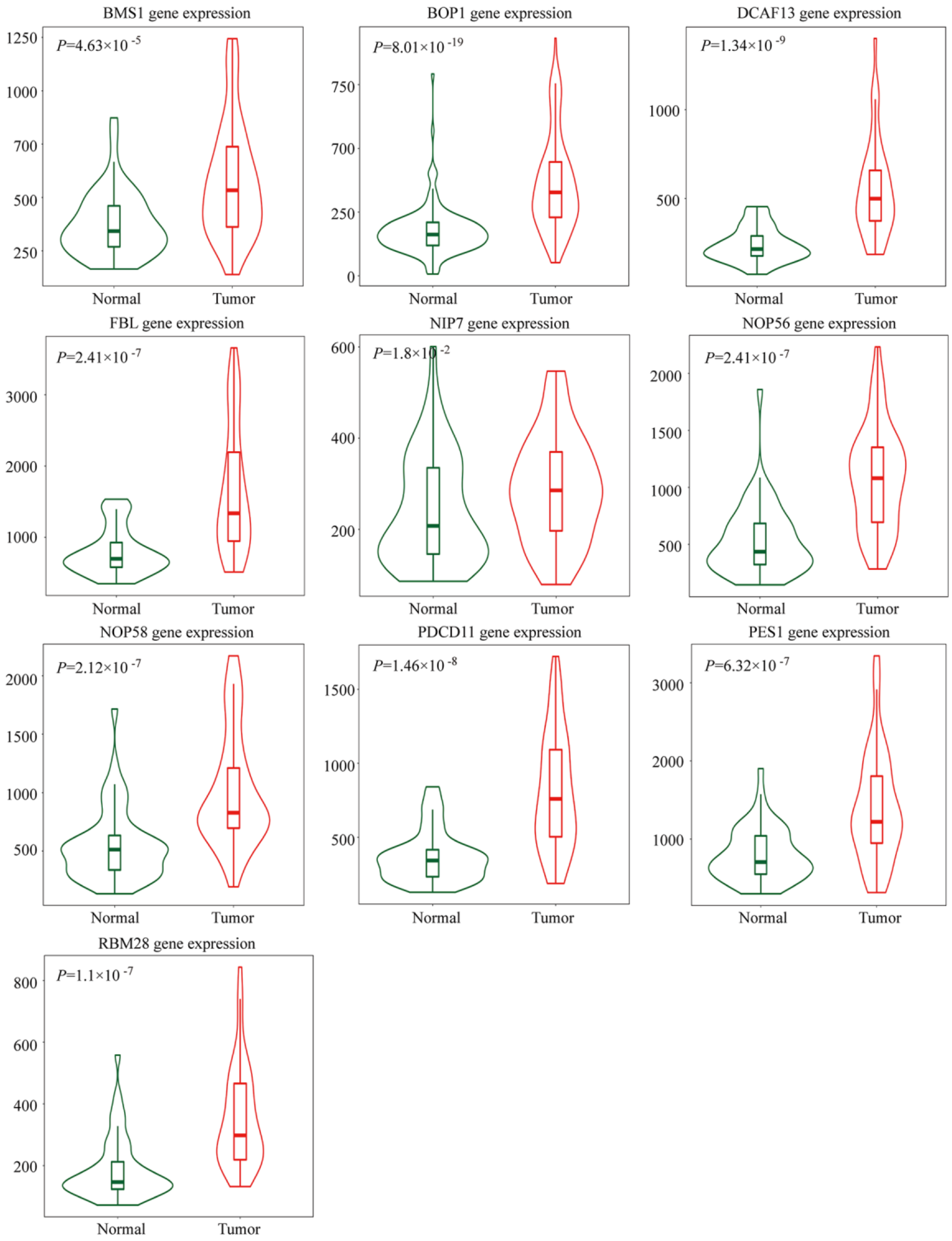

Supplementary Figure 6. The expressions of 10 potential hub genes. BMS1, DCAF13, FBL, NIP7, NOP56, NOP58, PDCD11, PES1, RBM28 (RNA-seq); BOP1 (gene chip).

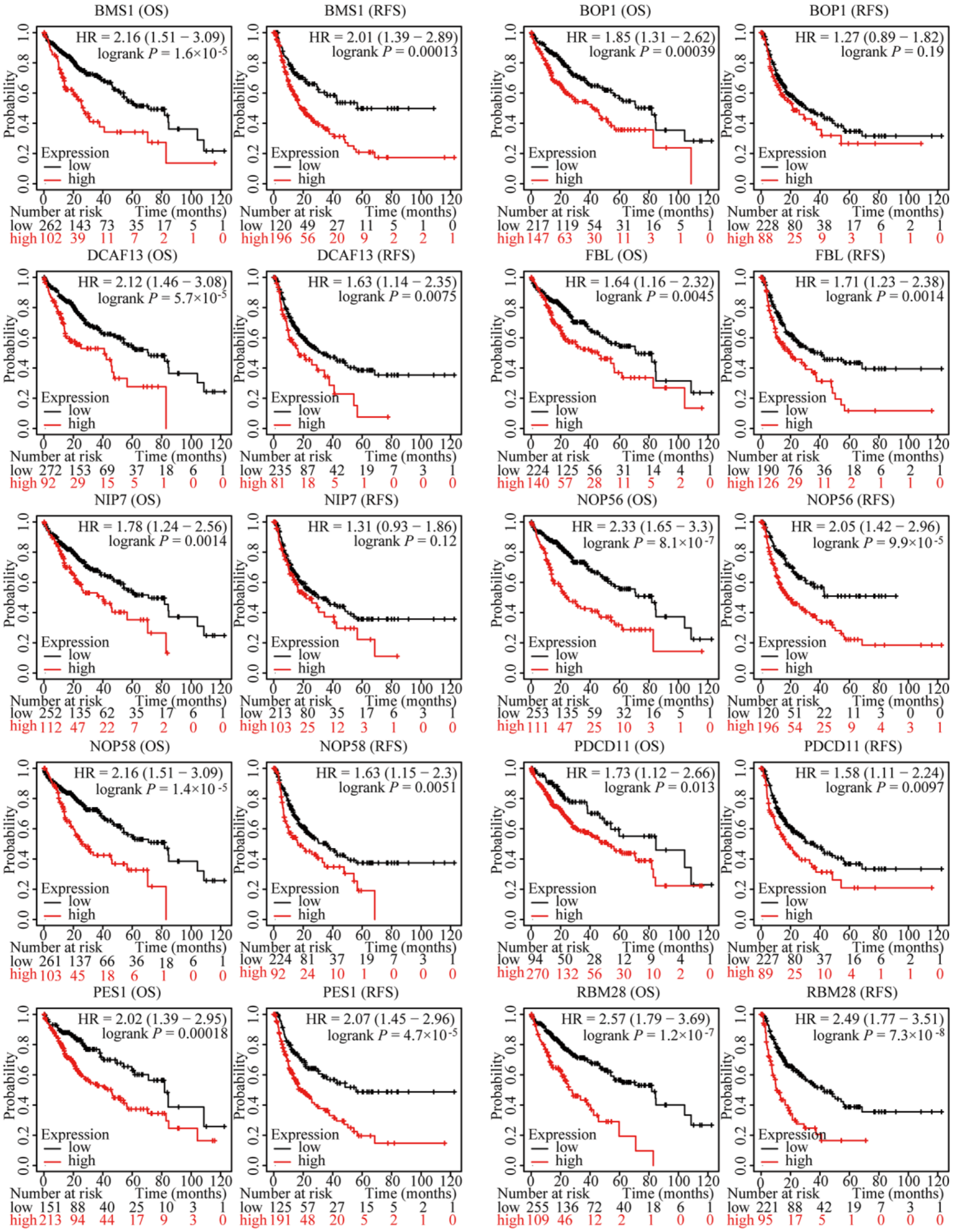

Supplementary Figure 7. The OS and RFS of 10 potential hub genes.

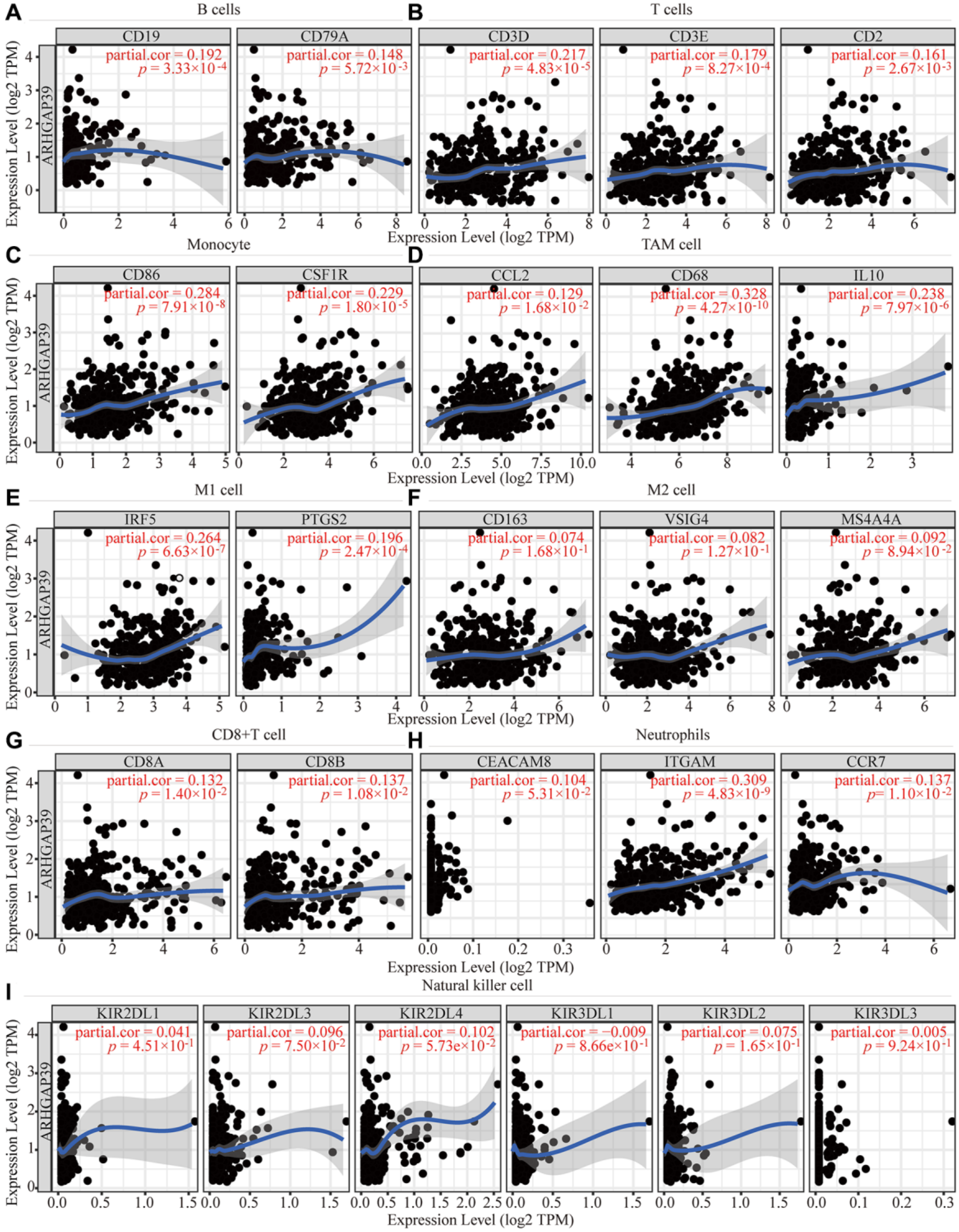

Supplementary Figure 8. Correlation of ARHGAP39 expression and the marker genes of infiltrating immune cells. The scatter plots showed a correlation between ARHGAP39 expression and the gene markers of (**A**) B cell (CD19, CD79A); (**B**) T cell (CD3D, CD3E, CD2); (**C**) Monocyte (CD86, CSF1R); (**D**) TAM cell (CCL2, CD68, IL10); (**E**) M1 cell (IRF5, PTGS2); (**F**) M2 cell (CD163, VSIG4, MS4A4A); (**G**) CD8+T cell (CD8A, CD8B) (**H**) Nutrophils (CEACAM8, ITGAM, CCR7); (**I**) Natural killer cell (KIR2DL1, KIR2DL3, KIR2DL4, KIR3DL1, KIR3DL2, KIR3DL3).

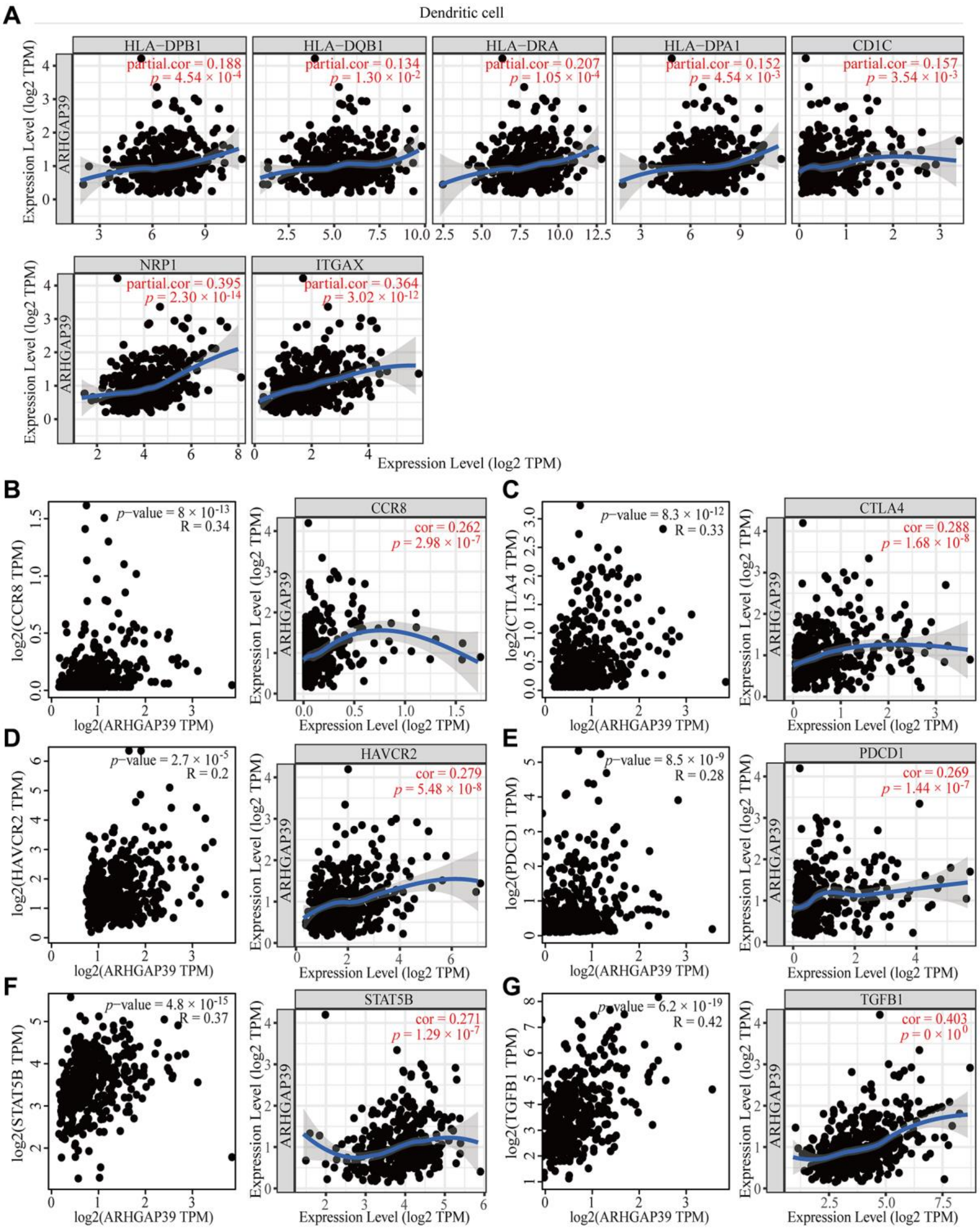

Supplementary Figure 9. Correlation of ARHGAP39 expression and T-cell checkpoint in HCC. (**A**) Dendritic cell (HLA-DPB1, HLA-DQB1, HLA-DRA, HLA-DPA1, CD1C, NRP1, ITGAX). (**B**) CCR8; (**C**) CTLA4; (**D**) HAVCR2; (**E**) PDCD1; (**F**) STAT5B; (**G**) TGFB1.

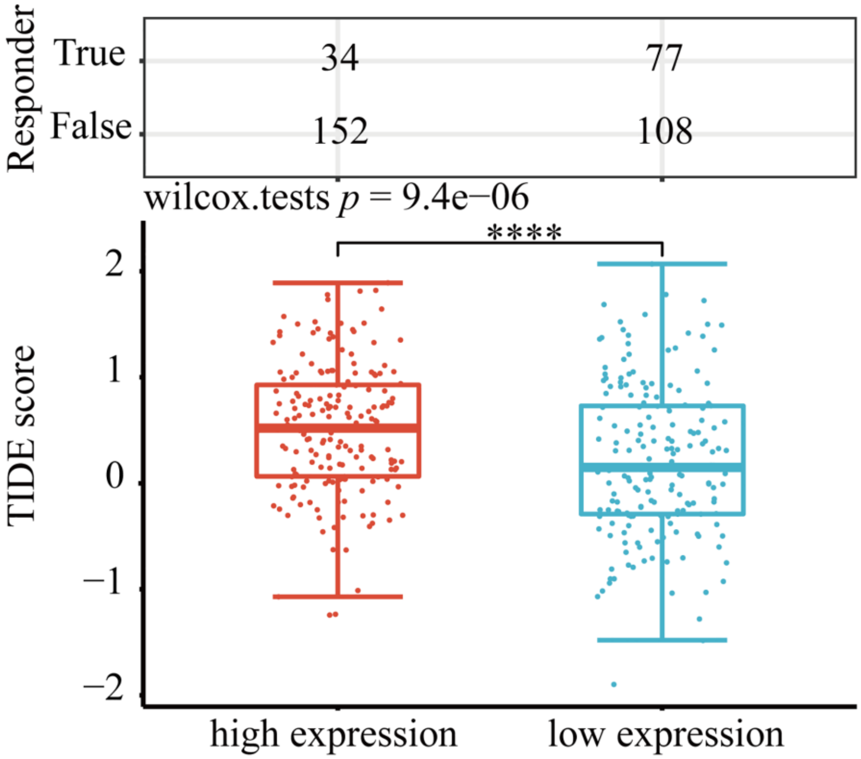

Supplementary Figure 10. Predict response to immunotherapy with different expressions of ARHGAP39. Statistical table of immune response of samples in different groups in the prediction results. The distribution of immune response scores in different groups in the prediction results.
