# Supplementary material for: Biological function analysis of ARHGAP39 as an independent prognostic biomarker in hepatocellular carcinoma
Source: Aging (Albany NY). 2023 Apr 5;15(7):2631–66. doi: 10.18632/aging.204635 (PMC10120899; doi:10.18632/aging.204635)
Supplement: Supplementary Tables [file aging-15-204635-s002.pdf]

SUPPLEMENTARY TABLES

Supplementary Table 1. Logistic analysis of the association between ARHGAP39 expression and clinical characteristics.

Clinical characteristics	Total (*N*)	0dds ratio in ARHGAP39 expression	*p* value
Age (>60 vs. ≤60)	370	0.82 (0.55–1.24)	0.349
Gender (Female vs. Male)	371	0.94 (0.61–1.45)	0.767
Grad (III vs. I)	366	1.97 (1.04–3.82)	0.041
Stage (II vs. I)	347	2.37 (1.40–4.07)	0.001
T (T2 vs. T1)	368	2.55 (1.54–4.29)	<0.001
M (M1 vs. M0)	270	0.33 (0.02–2.60)	0.338

Abbreviations: T: tumor; M: metastasis. Bold values indicate *P*-values < 0.05.

Supplementary Table 2. Univariate and multivariate COX regression analysis of factors associated with OS in HCC patients.

Variable	Univariate analysis			Multivariate analysis		
	HR	95%CI	*p* value	HR	95%CI	*p* value
**age**	1.007	0.990–1.024	0.441	1.004	0.986–1.021	0.688
**gender**	0.839	0.536–1.314	0.443	0.953	0.590–1.538	0.844
**grade**	1.073	0.795–1.449	0.645	1.16	0.839–1.602	0.369
**stage**	1.809	1.426–2.294	**<0.001**	1.165	0.473–2.867	0.74
**T**	1.767	1.415–2.207	**<0.001**	1.478	0.640–3.410	0.36
**M**	3.892	1.223–12.386	0.021	1.368	0.377–4.960	0.634
**ARHGAP39**	1.044	1.018–1.071	**<0.001**	1.028	1.000–1.057	**0.046**

Abbreviations: OS: overall survival; HR: hazard ratio; CI: confidence interval; T: tumor; M: metastasis. Bold values indicate *P*-values < 0.05.

Supplementary Table 3. Correlation analysis between ARHGAP39 and gene markers of different types of immune cells in TIMER.

Description	Gene markers	LIHC			
		None		Purity	
		Cor	*p*	Cor	*p*
**B cell**	CD19	0.21514202	**2.93E-05**	0.192090159	**0.000332661**
	CD79A	0.148788854	**0.004076138**	0.148486759	**0.005721239**
**T cell (general)**	CD3D	0.213994083	**3.37E-05**	0.216959705	**4.83E-05**
	CD3E	0.180086338	**0.000500732**	0.179202019	**0.000827122**
	CD2	0.160636795	**0.00193215**	0.161237177	**0.002667573**
**CD8+ T cell**	CD8A	0.150432042	**0.003711251**	0.132156146	**0.014027176**
	CD8B	0.142911461	**0.005823665**	0.137083903	**0.010802481**
**Monocyte**	CD86	0.277924364	**5.97E-08**	0.284117941	**7.91E-08**
	CSF1R	0.230041806	**8.05E-06**	0.228640291	**1.80E-05**
**TAM**	CCL2	0.162258981	**0.001735624**	0.128655402	**0.016805309**

	CD68	0.321517283	**2.89E-10**	0.327995094	**4.27E-10**
	IL10	0.247848996	**1.34E-06**	0.237844512	**7.97E-06**
**M1**	IRF5	0.274927931	**7.38E-08**	0.263881801	**6.63E-07**
	PTGS2	0.20591226	**6.45E-05**	0.196149212	**0.000246605**
	CD163	0.097940966	0.059477969	0.074437714	0.167735766
**M2**	VSIG4	0.10585096	**0.041621036**	0.082240565	0.127361276
	MS4A4A	0.11255167	**0.030245164**	0.091591547	0.089387909
	CEACAM8	0.106011904	**0.041271004**	0.104208391	0.053134243
**Neutrophils**	ITGAM	0.312210689	**9.70E-10**	0.308513525	**4.83E-09**
	CCR7	0.150896876	**0.003575499**	0.136724629	**0.011013188**
	KIR2DL1	0.072387638	0.164102562	0.040710738	0.451007396
	KIR2DL3	0.096335302	0.063796314	0.095981222	0.075008149
	KIR2DL4	0.114583645	**0.027323668**	0.102449497	0.057300233
**Natural killer cell**	KIR3DL1	0.002423069	0.962900439	-0.00909819	0.866283113
	KIR3DL2	0.072187225	0.165276271	0.074896197	0.165125921
	KIR3DL3	0.030764946	0.554711456	0.00518176	0.92360138
	HLA-DPB1	0.200793586	**0.000101668**	0.187773473	**0.000454351**
	HLA-DQB1	0.146143109	**0.004828004**	0.133630038	**0.012983888**
	HLA-DRA	0.216961627	**2.61E-05**	0.207261438	**0.000105349**
**Dendritic cell**	HLA-DPA1	0.17321831	**0.000819735**	0.152444383	**0.004541751**
	CD1C	0.17643679	**0.000640722**	0.156632616	**0.00353657**
	NRP1	0.42678733	**0**	0.395483231	**2.30E-14**
	ITGAX	0.343045846	**1.48E-11**	0.363970112	**3.02E-12**

Supplementary Table 4. Correlation analysis between ARHGAP39 and gene markers of different types of T cells in TIMER.

**Description**	**Gene markers**	**LIHC**			
		**None**		**Purity**	
		**Cor**	***p***	**Cor**	***p***
	TBX21	0.112938835	**0.02963177**	0.109674947	**0.04176303**
	STAT4	0.145706249	**0.004957276**	0.139635111	**0.009406142**
**Th1**	STAT1	0.294466521	**8.74E-09**	0.290923298	**3.72E-08**
	TNF	0.29154934	**1.06E-08**	0.309920364	**4.08E-09**
	IFNG	0.186421793	**0.000305821**	0.190956582	**0.000361277**
	HAVCR2	0.27868176	**5.48E-08**	0.291466867	**3.50E-08**
	IFNG	0.186421793	**0.000305821**	0.190956582	**0.000361277**
**Th1-like**	CXCR3	0.200356256	**0.000105328**	0.198372339	**0.000208781**
	BHLHE40	0.109962941	**0.034277494**	0.102484878	0.057213841
	CD4	0.077590433	0.135729075	0.048651285	0.367635364
**Th2**	STAT6	0.214683485	**3.05E-05**	0.190774558	**0.00036608**
	STAT5A	0.333461975	**4.37E-11**	0.317750581	**1.56E-09**
**Treg**	FOXP3	0.015020434	0.773075802	0.002180377	0.967812681

	CCR8	0.262287199	**2.98E-07**	0.267597268	**4.54E-07**
	TGFB1	0.403280091	**0**	0.423510722	**1.90E-16**
**Tfh**	BCL6	0.310716107	**1.17E-09**	0.293735496	**2.71E-08**
	CXCR5	0.241416379	**2.55E-06**	0.24604105	**3.75E-06**
**Th17**	STAT3	0.237365023	**4.04E-06**	0.219418931	**3.94E-05**
	IL17A	0.092479537	0.075225825	0.090611705	0.092881868
**Resting Treg**	FOXP3	0.015020434	0.773075802	0.002180377	0.967812681
	IL2RA	0.276903263	**5.90E-08**	0.275928797	**1.91E-07**
	FOXP3	0.015020434	0.773075802	0.002180377	0.967812681
**Effector Treg T-cell**	CCR8	0.262287199	**2.98E-07**	0.267597268	**4.54E-07**
	TNFRSF9	0.351897636	**2.96E-12**	0.347909952	**2.98E-11**
	CX3CR1	0.223066854	**1.52E-05**	0.213036153	**6.65E-05**
**Effector T-cell**	FGFBP2	-0.016050855	0.757977418	-0.034536459	0.52259598
	FCGR3A	0.167977873	**0.001179952**	0.153134883	**0.004360053**
**Naïve T-cell**	CCR7	0.150896876	**0.003575499**	0.136724629	**0.011013188**
	SELL	0.251134449	**1.04E-06**	0.245994551	**3.77E-06**
	DUSP4	0.312030211	**9.93E-10**	0.317758622	**1.56E-09**
**Effector memory T-cell**	GZMK	0.030523317	0.557826299	0.004254693	0.937238659
	GZMA	0.031365195	0.547010504	0.013491075	0.802829002
	CD69	0.130323928	**0.011988954**	0.110082966	**0.041003556**
**Resident memory T-cell**	CXCR6	0.10658075	**0.040188098**	0.093963405	0.081365678
	MYADM	0.454977593	**0**	0.448496853	**1.78E-18**
**General**	CCR7	0.150896876	**0.003575499**	0.136724629	**0.011013188**
**Memory T-cell**	SELL	0.251134449	**1.04E-06**	0.245994551	**3.77E-06**
	IL7R	0.112419415	**0.030394509**	0.091707856	0.088980262
	HAVCR2	0.27868176	**5.48E-08**	0.291466867	**3.50E-08**
**Exhausted T-cell**	LAG3	0.135820622	**0.008851065**	0.130221706	**0.015507838**
	CXCL13	0.082608948	0.112171638	0.081320723	0.131687351
	LAYN	0.268990711	**1.60E-07**	0.249025425	2.83E-06
